# *Lactobacillus rhamnosus* HDB1258 modulates gut microbiota-mediated immune response in mice with or without lipopolysaccharide-induced systemic inflammation

**DOI:** 10.1186/s12866-021-02192-4

**Published:** 2021-05-13

**Authors:** Sang-Kap Han, Yeon-Jeong Shin, Dong-Yeon Lee, Kyung Min Kim, Seo-Jin Yang, Du Seong Kim, Ji-Whi Choi, Seunghun Lee, Dong-Hyun Kim

**Affiliations:** 1 Neurobiota Research Center, College of Pharmacy, Kyung Hee University, 26, Kyungheedae-ro, Dongdaemun-gu, Seoul, 02447 South Korea; 2 HYUNDAI BIOLAND Co., Ltd., Ansan, 15407 South Korea

**Keywords:** *Lactobacillus rhamnosus* HDB1258, Immunomodulation, Immune enhancement, Inflammation, Gut microbiota

## Abstract

**Background:**

Gut microbiota closely communicate in the immune system to maintain a balanced immune homeostasis in the gastrointestinal tract of the host. Oral administration of probiotics modulates gut microbiota composition. In the present study, we isolated *Lactobacillus rhamnosus* HDB1258, which induced tumor necrosis factor (TNF)-α and interleukin (IL)-10 expression in macrophages, from the feces of breastfeeding infants and examined how HDB1258 could regulate the homeostatic immune response in mice with or without lipopolysaccharide (LPS)-induced systemic inflammation.

**Results:**

Oral administration of HDB1258 significantly increased splenic NK cell cytotoxicity, peritoneal macrophage phagocytosis, splenic and colonic TNF-α expression, TNF-α to IL-10 expression ratio, and fecal IgA level in control mice, while Th1 and Treg cell differentiation was not affected in the spleen. However, HDB1258 treatment significantly suppressed peritoneal macrophage phagocytosis and blood prostaglandin E2 level in mice with LPS-induced systemic inflammation. Its treatment increased LPS-suppressed ratios of Treg to Th1 cell population, Foxp3 to T-bet expression, and IL-10 to TNF-α expression. Oral administration of HDB1258 significantly decreased LPS-induced colon shortening, myeloperoxidase activity and NF-κB^+^/CD11c^+^ cell population in the colon, while the ratio of IL-10 to TNF-α expression increased. Moreover, HDB1258 treatment shifted gut microbiota composition in mice with and without LPS-induced systemic inflammation: it increased the Cyanobacteria and PAC000664_g (belonging to Bacteroidetes) populations and reduced Deferribacteres and EU622763_s group (belonging to Bacteroidetes) populations. In particular, PAC001066_g and PAC001072_s populations were negatively correlated with the ratio of IL-10 to TNF-α expression in the colon, while the PAC001070_s group population was positively correlated.

**Conclusions:**

Oral administered HDB1258 may enhance the immune response by activating innate immunity including to macrophage phagocytosis and NK cell cytotoxicity in the healthy host and suppress systemic inflammation in the host with inflammation by the modulation of gut microbiota and IL-10 to TNF-α expression ratio in immune cells.

**Supplementary Information:**

The online version contains supplementary material available at 10.1186/s12866-021-02192-4.

## Background

Gut microbiota closely communicate in the immune system to maintain a balanced immune homeostasis in the gastrointestinal tract of the host [1, 2]. The alteration of gut microbiota by exposure to gastrointestinal environmental factors such as stress, pathogens, and probiotics cause the immune system of the gastrointestinal tract to fluctuate [3]. This exposure stimulates the secretion of proinflammatory and anti-inflammatory cytokines such as tumor necrosis factor (TNF)-α and interleukin (IL)-10 in the immune cells, > 70% of which are located in the gut [4, 5]. These secreted cytokines regulate the immune system consisting of innate and adaptive immune systems. The activation of innate immune cells, which consist of phagocytic leukocytes and natural killer (NK) cells, by microbes stimulates the adaptive immune cells, which consist of T and B cells, through the regulation of cytokine expression [5, 6]. The secretion of cytokines such as TNF-α and IL-10 in innate immune cells by the stimulation of pathogens promotes the differentiation of naïve T cells into effector T cells such as helper T (Th) and regulatory T (Treg) cells [7, 8]. A wide imbalance between innate and adaptive immune systems, such as hyperimmunopotentiation and immunosuppression, is a high-risk factor for the outbreak of infectious diseases, chronic inflammation, autoimmunity, and cancers [9, 10]. Therefore, regulating the immune response in the gut may be beneficial for the therapy of peripheral and systemic immune disorders.

Probiotics including Lactobacilli, which are commonly found in fermented foods such as yogurt and kimchi and the gut microbiota of humans and animals, exhibit the beneficial physiological activities including the protection from pathogen infection and modulation of the immune system via the gastrointestinal tract [11–13]. *Lactobacillus reuteri* alleviates ampicillin- or lipopolysaccharide-induced colitis and gut dysbiosis [14, 15]. TNF-α expression-inhibiting *Lactobacillus johnsonii* significantly alleviates 2,4,6-trinitrobenzenesulfonic acid- or immobilization stress-generated gut inflammation and disruption in mice [15, 16]. IL-6 expression-inhibitory *Lactobacillus reuteri* NK33 alleviates immobilization stress-generated gut inflammation and dysbiosis in mice [17]. TNF-α expression-inhibitory *Lactobacillus mucosae* NK41 alleviates *Escherichia coli*-generated gut inflammation and dysbiosis in mice [18]. IL-10 expression-inducing Lactobacilli alleviate high-fat diet-generated gut inflammation and microbiota alteration in mice [19]. However, TNF-α express-inducing probiotics potentiate the immune response in mice [20]. These results suggest that probiotics are able to mitigate hyperresponsive and hyporesponsive immune responses. However, how probiotics can homeostatically regulate the immune responses remains unclear.

Therefore, we selected a probiotic *Lactobacillus rhamnosus* HDB1258 from bacterial strains isolated from the feces of breastfeeding infants and examined its effects on the innate and adaptive immune responses in the spleen and colon of mice with or without LPS-induced systemic inflammation.

## Results

### Effect of HDB1258 on the expression of TNF-α and IL-10 in vitro

**Fig. 1:**
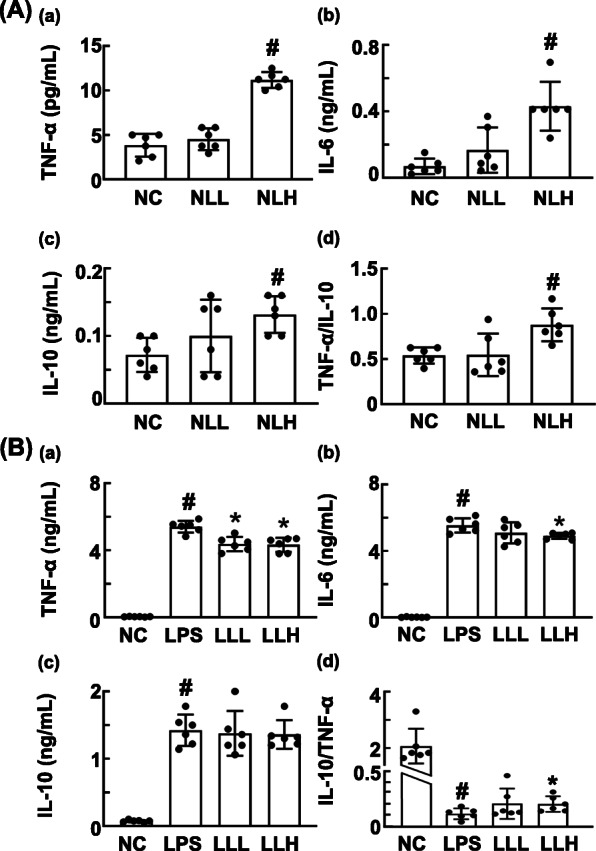
Effect of HDB1258 on the expression of proinflammatory cytokines in macrophages stimulated with or without LPS. **A** Effect on TNF-α **a**, IL-6 **b**, and IL-10 expression **c** and ratio of TNF-α to IL-10 expression **d** in macrophage cells. **B** Effect on TNF-α **a**, IL-6 **b**, and IL-10 expression **c** and ratio of IL-10 to TNF-α expression **d** in LPS-stimulated macrophage cells. Macrophage cells (1 × 10^6^/mL) isolated from peritoneal cavity were incubated with HDB1258 (LL, 1 × 10^4^ CFU/mL; LH, 1 × 10^5^ CFU/mL) in the absence or presence of LPS. Normal control group (NC) was treated with saline instead of LPS. Cytokine expression levels were assayed using ELISA kits. Data values were described as mean ± SD (*n* = 6). ^#^*p* < 0.05 vs. NC. ^*^p < 0.05 vs. group treated with LPS alone

To understand whether probiotics could homeostatically modulate immune response (immunopotentiation and immunosuppression), first we selected three probiotics, which induced the expression of TNF-α in macrophages, from the collection of bacterial strains isolated from the feces of breastfeeding infants. Of these, HDB1258 most potently induced TNF-α and IL-10 expression in macrophage (Supplementary Fig. S1). HDB1258 also induced the expression of IL-6, a proinflammatory cytokine, and IL-10, an anti-inflammatory cytokine, in macrophage cells (Fig. 1). HDB1258 increased the expression ratio of TNF-α to IL-10. However, HDB1258 suppressed the expression of TNF-α and IL-6 in LPS-stimulated macrophages and did not affect the expression of IL-10. Furthermore, it weakly increased the ratio of IL-10 to TNF-α expression in LPS-stimulated macrophages.

### HDB1258 potentiated the immune response including the innate and adaptive immune systems in mice

**Fig. 2:**
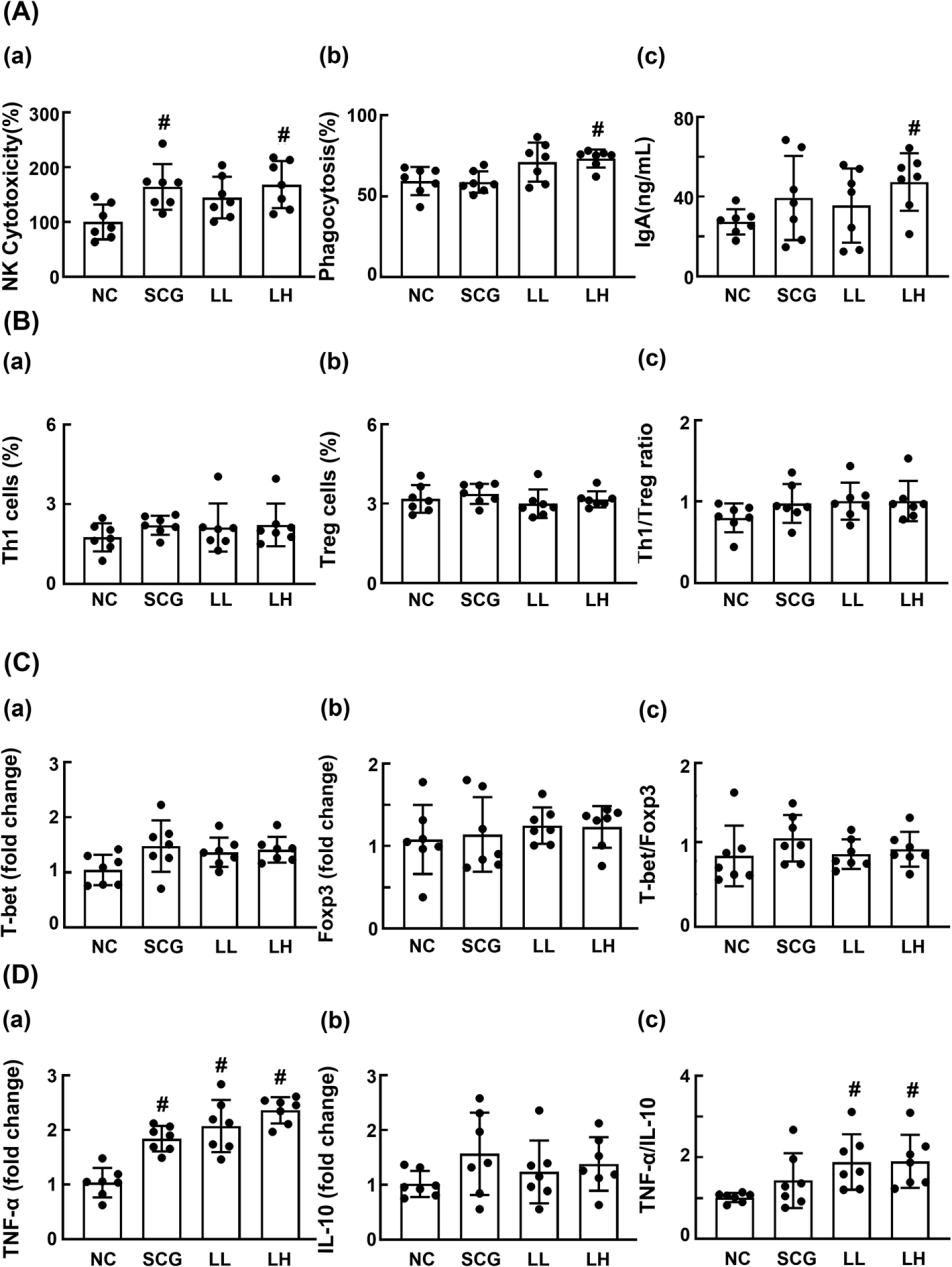
Effect of HDB1258 and *Saccharomyces cerevisiae* (SC) β-glucan on splenic NK cell cytotoxicity, peritoneal macrophage phagocytosis, fecal IgA level, splenic Th1 and Treg cell differentiation, splenic T-bet, Foxp3, TNF-α, and IL-10 expression in mice. **A** Effects on splenic NK cell cytotoxicity **a**, peritoneal macrophage phagocytosis **b**, and fecal IgA level **c**. **B** Effects on the differentiation of Th1 **a** and Treg cells **b** and ratio of Th1 to Treg cells **c**. **C** Effects on the expression of T cell transcription factors T-bet **a** and Foxp3 **b** and ratio of T-bet to Foxp3 expression **c**. **D** Effects on the expression of TNF-α **a** and IL-10 **b** and ratio of TNF-α to IL-10 expression. HDB1258 (LL, 1 × 10^8^ CFU/mouse/day and LH, 1 × 10^9^ CFU/mouse/day) or SC β-glucan (SCG, 50 mg/kg/day) was orally gavaged daily for 14 days. Normal control mice (NC) were treated with vehicle (saline) instead of test agents. Cytokine expression levels were assayed using qPCR. Data values were described as mean ± SD (*n* = 7). ^#^*p* < 0.05 vs. NC

First, to understand whether HDB1258 could activate the innate immune cells such as NK cells and macrophages, we orally gavaged HDB1258 or *Saccharomyces cerevisiae* (SC) β-glucan, which is known as an immunopotentiator [21], in mice and assayed the cytotoxicity of splenic NK cells against YAC-1 tumor cells and phagocytosis of peritoneal macrophages against *Candida albicans* (Fig. 2). Oral gavage of HDB1258 at a dose of 1 × 10^9^ CFU/mouse significantly increased splenic NK cell cytotoxicity and peritoneal macrophage phagocytic activity. Furthermore, oral HDB1258 increased the secretion of IgA into the feces. SC β-glucan at a dose of 50 mg/kg significantly increased the splenic NK cell activity, but not macrophage phagocytosis and IgA secretion.

Next, to understand whether HDB1258 could stimulate the adaptive immune cells such as T cells, we also examined the effect of HDB1258 on the CD4^+^IFNγ^+^ (Th1) and CD4^+^CD25^+^Foxp3^+^ (Treg) cell differentiation and their transcription factor expression in the spleen of mice. Oral gavage of HDB1258 or SC β-glucan did not affect Th1 and Treg cell differentiation and their transcription factor expression. To confirm these immune responses, we assayed TNF-α, a proinflammatory cytokine, and IL-10, an anti-inflammatory cytokine, in the spleen using qPCR. Treatment with HDB1258 or SC β-glucan increased significantly increased TNF-α expression. However, it did not significantly affect the expression of IL-10 in the spleen. As a result, oral gavage of HDB1258, but not SC β-glucan, increased the ratio of TNF-α to IL-10 expression in the spleen.

**Fig. 3:**
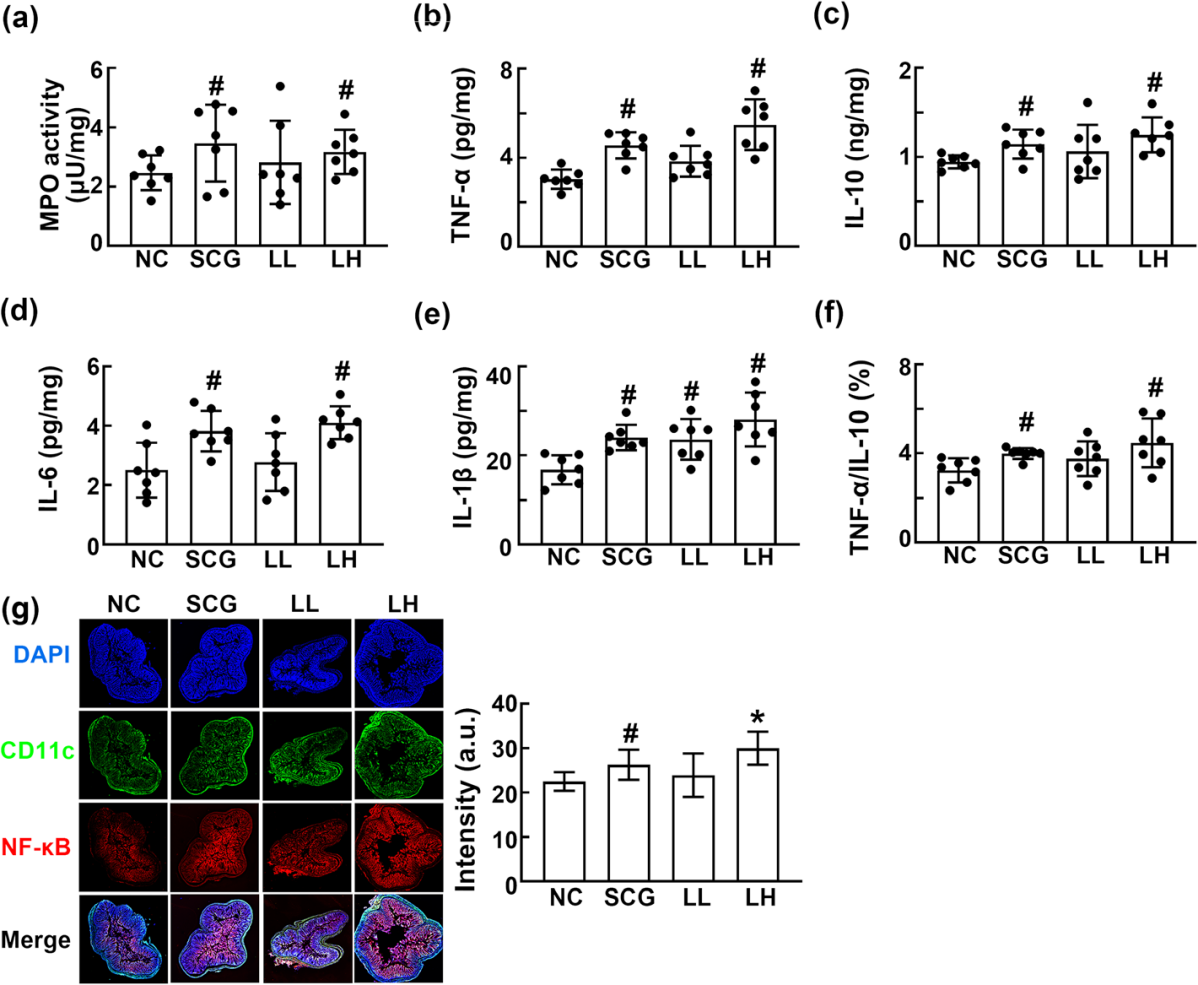
Effect of HDB1258 and SC β-glucan on the myeloperoxidase activity **a**, TNF-α **b**, IL-10 **c**, IL-6 **d**, and IL-1β expression **e**, ratio of TNF-α to IL-10 expression **f**, and NF-κB^+^CD11c^+^ cell population **g** in the colon of mice. HDB1258 (LL, 1 × 10^8^ CFU/mouse/day and LH, 1 × 10^9^ CFU/mouse/day) or SC β-glucan (SCG, 50 mg/kg/day) was orally gavaged daily for 14 days. Normal control mice (NC) were treated with vehicle (saline) instead of test agents. Cytokine expression levels were assayed using ELISA kits. Data values were described as mean ± SD (n = 7). ^#^*p* < 0.05 vs. NC

Oral gavage of HDB1258 at a dose of 1 × 10^9^ CFU/mouse or SC β-glucan at a dose of 50 mg/kg induced TNF-α, IL-1β, IL-6, and IL-10 expression in the in the colon (Fig. 3). In particular, it increased the ratio of TNF-α to IL-10 expression in the colon. They increased myeloperoxidase activity and NF-κB^+^/CD11c^+^ cell population.

### Effect of HDB1258 on gut microbiota composition in mice

**Fig. 4:**
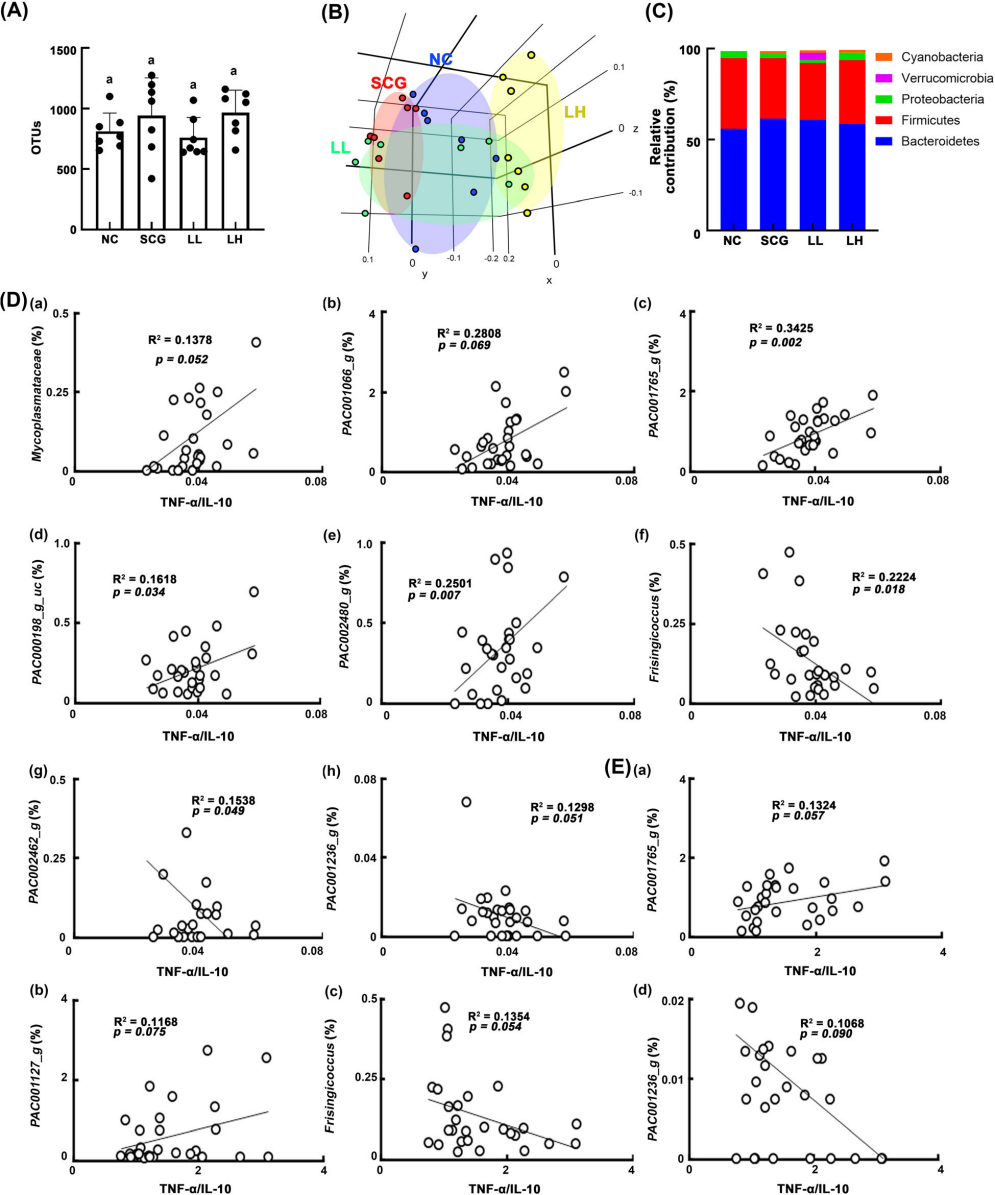
Effect of HDB1258 and SC β-glucan on the gut microbiota composition in mice. **A** Effects on α-diversity (OUT richness). **B** Effects on β-diversity. **C** Effects on the gut bacteria composition at the phylum level. **D** The correlation between gut microbiota (at the family and genus levels) and ratio of TNF-α to IL-10 expression in the colon. **E** The correlation between gut microbiota (at the family and genus levels) and TNF-α to IL-10 expression ratio in the spleen. HDB1258 (LL, 1 × 10^8^ CFU/mouse/day and LH, 1 × 10^9^ CFU/mouse/day) or SC β-glucan (SCG, 50 mg/kg/day) was orally gavaged daily for 14 days. Normal control group (NC) was treated with saline instead of test agents. Cytokine expression levels in the spleen were assayed using qPCR and those in the colon were using ELISA. Data values were described as mean ± SD (*n* = 7). ^#^*p* < 0.05 vs. NC

Gut microbiota are closely associated with gut immunity [1]. Probiotics significantly modulate the gut microbiota composition [12, 14]. Therefore, we examined whether the immunopotentiating effects of HDB1258 and SC β-glucan were associated with the composition of gut microbiota in mice (Fig. 4). Oral gavage of HDB1258 also modified the composition of gut microbiota in mice: it shifted β-diversity (principal coordinate analysis [PCoA]) while the α-diversity (operational taxonomic unit [OUT] richness) was not affected (Fig. 4a, b). HDB1258 treatment also increased the Cyanobacteria and Tenericutes populations and reduced the Firmicutes and Deferribacteres populations at the phylum level (Fig. 4c, Supplementary Tables S1, S2, S3). It also increased the Bacteroidaceae and FR888536_f (belonging to cyanobacteria) populations at the family level, PAC000664_g (belonging to Firmicutes), Paraprevotella, Muribaculaceae_uc populations at the genus level, and AB606242_s (belonging to Fimicutes), PAC001072_s (belonging to Bacteroidetes), and FJ880724_s (belonging to Bacteroidetes) population at the species level and reduced the Heliobacteriaceae, Deferribacteriaceae, and Coribacteriaceae populations at the family level, Prevotellaceae_uc, LLKB_g (belonging to Firmicutes), and Eubacterium_g6 populations at the genus level, and EU505186_s (belonging to Bacteroidetes) and AB626939_s (belonging to Firmicutes) populations at the species level. To determine whether gut microbiota are related to the immunopotentiating effects of HDB1258, we analyzed the correlation between the TNF-α to IL-10 expression ratio, which is the correlated with actual immune response in vivo [22, 23], and gut microbiota in mice treated with and without HDB1258 (Fig. 4d, Supplementary S2). Mycoplasmataceae (R = 0.371, *p* = 0.052), PAC001066_g (R = 0.530, *p* = 0.069), PAC001765_g (R = 0.585, *p* = 0.002), Mycoplasma_g10 (R = 0.364, *p* = 0.055), PAC001072_s (R = 0.055, *p* = 0.002), PAC001076_s (R = 0.604, *p* < 0.001), PAC002476_s (R = 0.382, *p* = 0.045), PAC002451_s (R = 0.654, *p* < 0.001), PAC000198_g_uc (R = 0.402, *p* = 0.034), and PAC002480_s (R = 0.500, *p* = 0.007) populations showed a positive correlation with the ratio of TNF-α to IL-10 expression in the colon. Frisingicoccus (R = − 0.472, *p* = 0.018), PAC002462_g (R = 0.-0.392, *p* = 0.049), PAC001236_g (R = − 0.360, *p* = 0.051), PAC001070_s group (R = − 0.355, *p* = 0.064) populations showed a negative correlation with the ratio of TNF-α to IL-10 expression in the colon. PAC001765_g (R = 0.364, *p* = 0.057), PAC001127_g (R = 0.342, *p* = 0.075), PAC001072_s (R = 0.498, *p* = 0.007), PAC001084_s (R = 0.322, *p* = 0.095), PAC001076_s (R = 0.385, *p* = 0.043), PAC002451_s (R = 0.454, *p* = 0.015), PAC001081_s group (R = 0.380, *p* = 0.046), PAC001114_s (R = 0.453, *p* = 0.015), PAC001095_s (R = 0.409, *p* = 0.031), PAC001113_s (R = 0.448, *p* = 0.017), and PAC002480_s (R = 0.383, *p* = 0.044) populations showed a positive correlation with the ratio of TNF-α to IL-10 expression in the spleen. Frisingicoccus (R = − 0.368, *p* = 0.054), PAC000197_g (R = − 0.303, *p* = 0.118), PAC001236_g (R = − 0.327, *p* = 0.090) populations showed a negative correlation with the ratio of TNF-α to IL-10 expression in the spleen.

### HDB1258 alleviated LPS-induced inflammation in mice

**Fig. 5:**
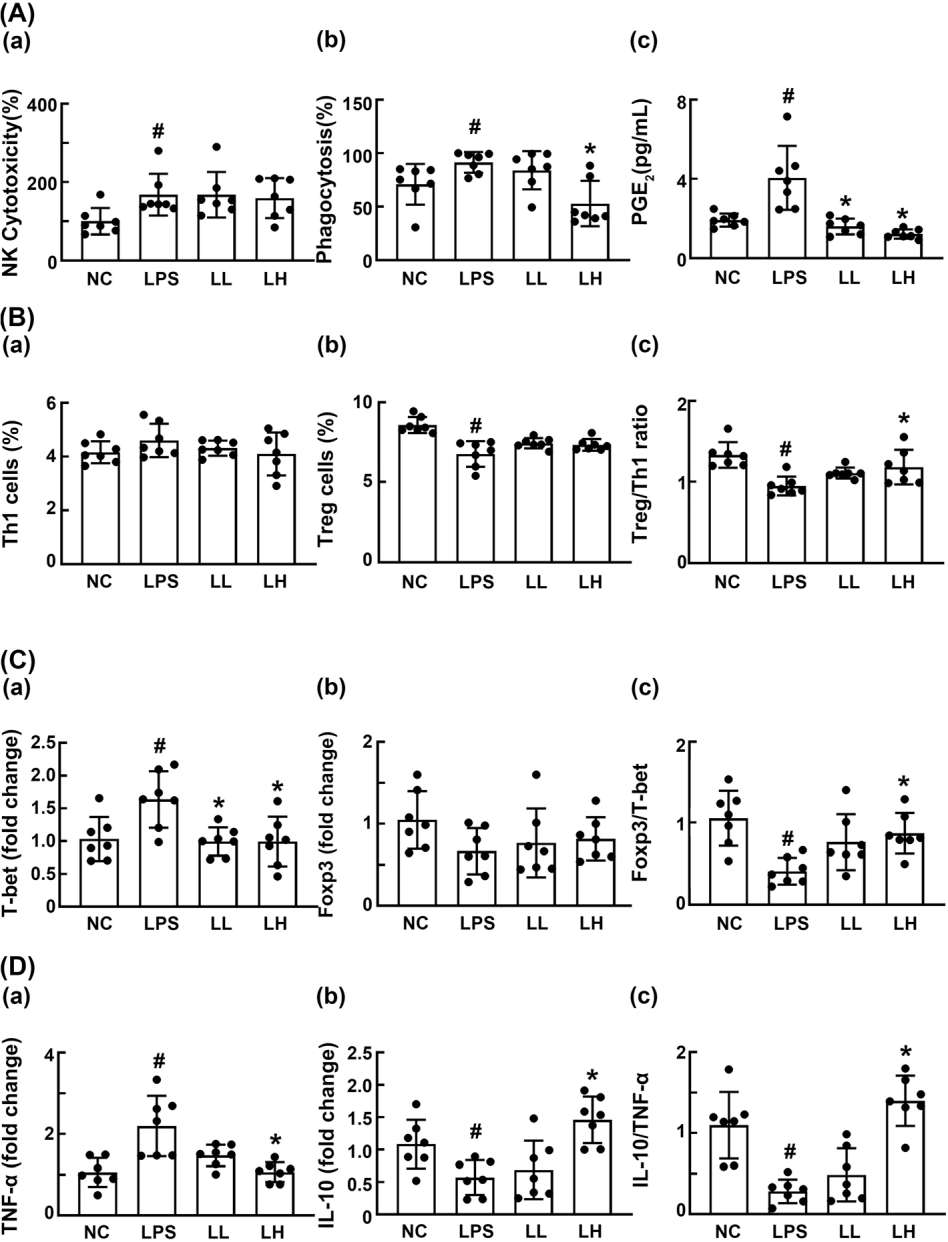
Effect of HDB1258 on splenic NK cell cytotoxicity, peritoneal macrophage phagocytosis, blood PGE2 level, splenic Th1 and Treg cell differentiation, splenic T-bet, Foxp3, TNF-α, and IL-10 expression in mice with LPS-induced systemic inflammation. **A** Effects on splenic NK cell cytotoxicity **a**, peritoneal macrophage phagocytosis **b**, and blood PGE2 level **c**. **B** Effects on the differentiation of Th1 **a** and Treg cells **b** and ratio of Th1 to Treg cells **c**. **C** Effects on the expression of T cell transcription factors T-bet **a** and Foxp3 **b** and ratio of T-bet to Foxp3 expression **c**. **D** Effects on the expression of TNF-α **a** and IL-10 **b** and ratio of TNF-α to IL-10 expression **C**. Mice was interaperitoneally injected with LPS (10 μg/kg/day) for 10 days. HDB1258 (LPS, vehicle; LL, 1 × 10^8^ CFU/mouse/day and LH, 1 × 10^9^ CFU/mouse/day) was orally gavaged daily for 14 days from the final injection of LPS. Normal control mice (NC) were treated with saline instead of LPS and test agents. Cytokine expression levels were assayed using qPCR. Data values were described as mean ± SD (*n* = 7). ^#^*p* < 0.05 vs. NC. ^*^p < 0.05 vs. group treated with LPS alone

Next, we examined the effect of HDB1258 on the innate immune response including the cytotoxicity of NK cell and phagocytosis of macrophages in LPS-stimulated mice (Fig. 5a). Intraperitoneal injection of LPS significantly increased NK cell cytotoxicity against YAC-1 tumor cells and peritoneal macrophage phagocytic activity against *Candida albicans*. However, HDB1258 at a dose of 1 × 10^9^ CFU/mouse significantly suppressed macrophage phagocytosis, not LPS-induced NK cell cytotoxicity. Furthermore, HDB1258 treatment suppressed the LPS-induced PGE2 level. Next, we examined the effect of HDB1258 on LPS-induced systemic inflammation in mice (Fig. 5b). Intraperitoneal injection of LPS significantly decreased the CD4^+^CD25^+^Foxp3^+^ (Treg) cell population in the spleen, while the CD4^+^IFNγ^+^ (Th1) cell population was not affected. Nevertheless, LPS treatment reduced the ratio of CD4^+^CD25^+^Foxp3^+^ to CD4^+^IFNγ^+^ cell population. Oral gavage of HDB1258 did not significantly affect the population of Th1 or Treg cells. However, HDB1258 treatment recovered LPS-suppressed ratio of Treg to Th1 cell population. LPS treatment increased the expression of Th1 transcription factor T-bet in the spleen while the expression of Treg cell transcription factor Foxp3 was weakly, but not significantly, suppressed. Nevertheless, the ratio of Foxp3 to T-bet expression was increased by LPS treatment. However, oral gavage of HDB1258 significantly reduced LPS-induced T-bet expression, while the Foxp3 expression was not affected. Nevertheless, HDB1258 treatment recovered the LPS-suppressed ratio of Treg to Th1 cell population. LPS treatment increased the TNF-α expression, while IL-10 expression decreased. Moreover, its treatment decreased the ratio of IL-10 to TNF-α expression. Oral gavage of HDB1258 significantly suppressed LPS-induced TNF-α expression and induced LPS-suppressed IL-10 expression. Furthermore, HDB1258 treatment increased the LPS-suppressed ratio of IL-10 to TNF-α expression.

**Fig. 6:**
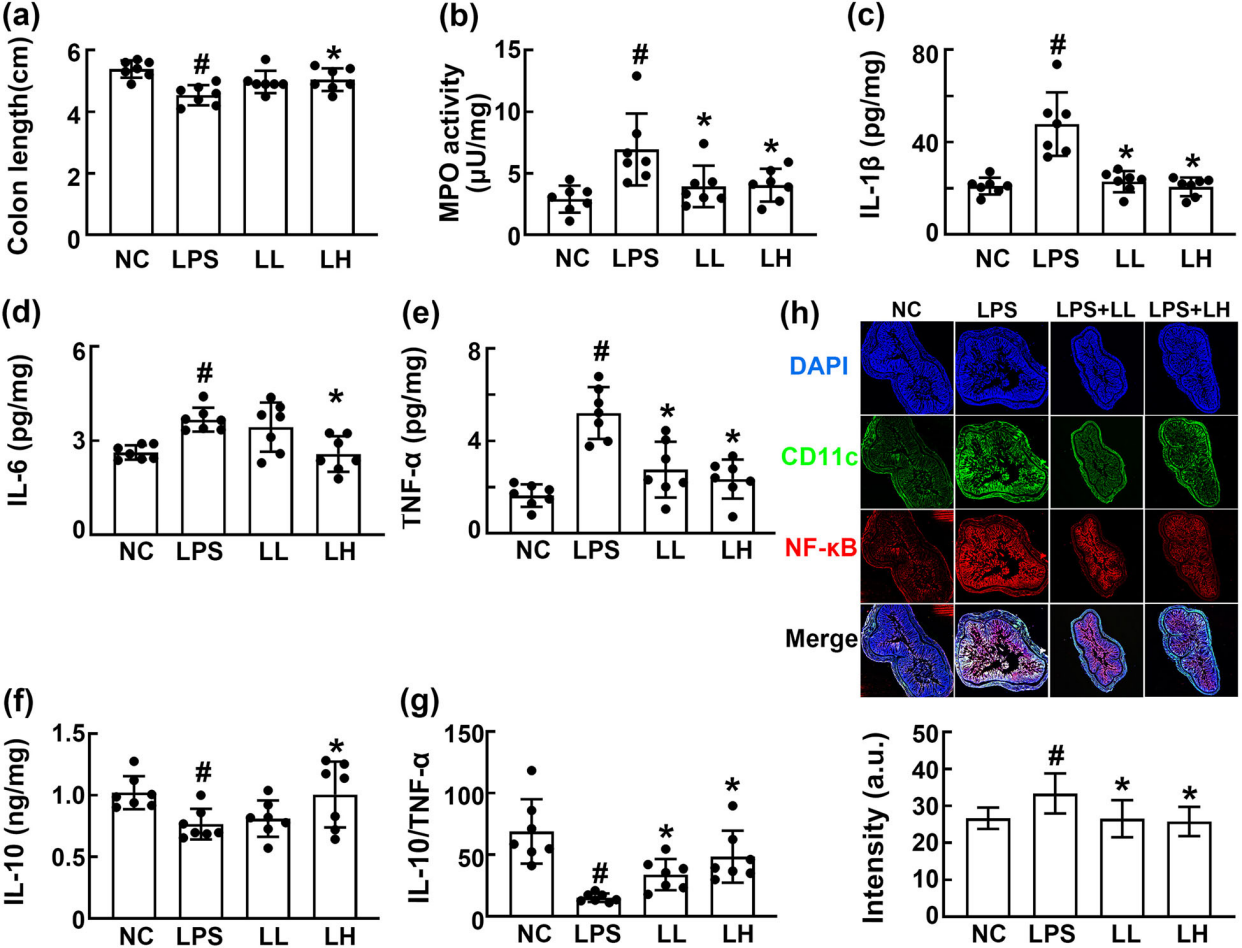
Effect of HDB1258 on the LPS-induced colitis in mice with LPS-induced systemic inflammation. on the colon length **a**, myeloperoxidase (MPO) activity **b**, IL-1β **c**, IL-6 **d**, TNF-α **e**, and IL-10 expression **f**, ratio of IL-10 to TNF-α expression **g**, and NF-κB^+^CD11c^+^ cell population **h**. Mice was intraperitoneally injected with LPS (10 μg/kg/day) for 10 days. HDB1258 (LPS, vehicle; LL, 1 × 10^8^ CFU/mouse/day and LH, 1 × 10^9^ CFU/mouse/day) was orally gavaged daily for 14 days from the final injection of LPS. Normal control mice (NC) were treated with saline instead of LPS and test agents. Cytokine expression levels were assayed using ELISA kits. Data values were described as mean ± SD (n = 7). ^#^*p* < 0.05 vs. NC. ^*^p < 0.05 vs. group treated with LPS alone

Furthermore, we examined the effect of HDB1258 on the colitis in mice with LPS-induced systemic inflammation (Fig. 6). Intraperitoneal injection of LPS induced colitis: it induced colon shortening, IL-1β, IL-6, and TNF-α expression and suppressed IL-10 expression in the colon. In particular, LPS treatment increased the TNF-α to IL-10 expression ratio and NF-kB^+^CD11c^+^ cell population in the colon. However, oral gavage of HDB1258 significantly inhibited LPS-induced colon shortening and myeloperoxidase activity and NF-κB^+^/CD11c^+^ cell population. Furthermore, its treatment increased the ratio of IL-10 to TNF-α expression in the colon.

### HDB1258 partially modified LPS-disturbed gut microbiota composition in mice

**Fig. 7:**
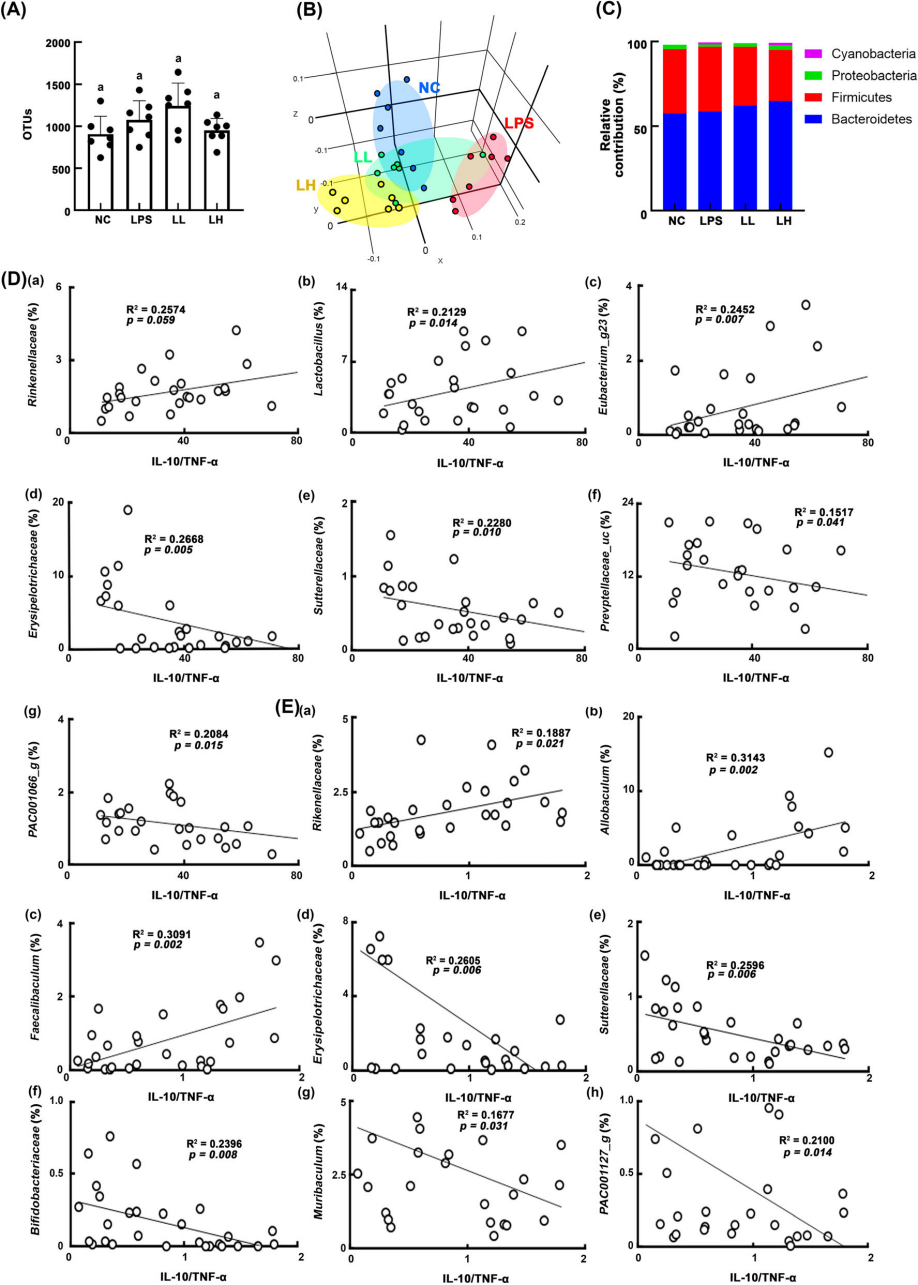
Effect of HDB1258 on the composition of gut microbiota in mice with LPS-induced systemic inflammation. **A** Effects on α-diversity (OUT richness). **B** Effects on β-diversity. **C** Effects on the gut bacteria composition at the phylum level. **D** The correlation between gut microbiota (at the family and genus levels) and ratio of IL-10 to TNF-α expression in the colon. **E** The correlation between gut microbiota (at the family and genus levels) and ratio of IL-10 to TNF-α expression in the spleen. Mice was intraperitoneally injected with LPS (10 μg/kg/day) for 10 days. HDB1258 (LPS, vehicle; LL, 1 × 10^8^ CFU/mouse/day and LH, 1 × 10^9^ CFU/mouse/day) was orally gavaged daily for 14 days from the final injection of LPS. Normal control mice (NC) were treated with vehicle (saline) instead of LPS and test agents. Cytokine expression levels in the spleen were assayed using qPCR and those in the colon were using ELISA. Data values were described as mean ± SD (n = 7). ^#^*p* < 0.05 vs. NC. ^*^p < 0.05 vs. group treated with LPS alone

Next, we examined whether the anti-inflammatory effects of HDB1258 were associated with the gut microbiota composition in mice (Fig. 7). Exposure to LPS also caused gut microbiota alteration in mice: it shifted β-diversity (PCoA) while the α-diversity (OTU richness) was not affected (Fig. 7a, b). Furthermore, it increased cyanobacteria population and reduced the Tenericutes, Verrucomicrobia, and Deferribacteres populations (Fig. 7c, Supplementary Tables S4, S5, S6). Oral gavage of HDB1258 changed LPS-shifted β-diversity in the gut microbiota, while the α-diversity was weakly, but not significantly, affected. HDB1258 treatment also increased LPS-suppressed Tenericutes, Verrucomicrobia, and Deferribacteres populations at the phylum level, Lachnospiraceae, Rikenellaceae, Helicobacteriaceae, Akkermansiaceae, Odoribacteriaceae, and Deferribacteraceae populations at the family level, KE159538_g (belonging to Firmicutes), PAC000664_g (belonging to Bacteroidetes), and Muribaculum populations at the genus level, and PAC001696_s (Firmicutes), PAC001120_s (belonging to Firmicutes), and PAC001077_s (belonging to Bacteroidetes) populations at the species level and reduced LPS-induced Erysipelotrichaceae population at the family level, Ruminococcus population at the genus level, and EU622763_s group (belonging to Bacteroidetes) population at the species level. To determine whether gut microbiota are related to the anti-inflammatory effect of HDB1258, we analyzed the correlation between the ratio of IL-10 to TNF-α expression and gut microbiota in mice with LPS-induced systemic inflammation (Fig. 7d, Supplementary Fig. S3). Rikenellaceae (R = 0.507, *p* = 0.059), Lactobacillus (R = 0.462, *p* = 0.014), Eubacterium_g23 (R = 0.495, *p* = 0.007), PAC001070_s group (R = 0.418, *p* = 0.027), *Lactobacillus murinus* group (R = 0.449, *p* = 0.017), *Lactobacillus reuteri* group (R = 0.412, *p* = 0.029), PAC001982_s (R = 0.434, *p* = 0.021), PAC000661_g_uc (R = 0.632, *p* < 0.001) populations showed a positive correlation with the ratio of IL-10 to TNF-α expression in the colon. Erysipelotrichaceae (R = − 0.517, *p* = 0.005), Sutterellaceae (R = − 0.478, *p* = 0.010), Prevotellaceae_uc (R = − 0.389, *p* = 0.041), PAC001066_g (R = − 0.457, *p* = 0.015), PAC001072_s (R = − 0.362, *p* = 0.059), PAC001066_s (R = − 0.475, *p* = 0.011), PAC002478_s (R = − 0.488, *p* = 0.009), PAC001756_s (R = − 0.614, *p* < 0.001) populations showed a negative correlation with the ratio of IL-10 to TNF-α expression in the colon. Rikenellaceae (R = 0.434, *p* = 0.021), Odoribacteraceae (R = 0.406, *p* = 0.032), PAC000661_g (R = 0.394, *p* = 0.038), Allobaculum (R = 0.561, *p* = 0.002), Faecalibaculum (R = 0.556, *p* = 0.002), *Bacteroides acidifaciens* group (R = 0.412, *p* = 0.029), PAC001081_s group (R = 0.420, *p* = 0.026), and FJ880578_s (R = 0.537, *p* = 0.003) populations showed a positive correlation with the ratio of IL-10 to TNF-α expression in the spleen. Erysipelotrichaceae (R = − 0.510, *p* = 0.006), Sutterellaceae (R = − 0.510, *p* = 0.006), Bifidobacteriaceae (R = − 0.489, *p* = 0.008), Muribaculum (R = − 0.410, *p* = 0.031), PAC001127_g (R = 0.458, *p* = 0.014), PAC001084_s (R = − 0.388, *p* = 0.042), and PAC001756_s (R = − 0.531, *p* = 0.004) populations showed a negative correlation with the ratio of IL-10 to TNF-α expression in the spleen.

## Discussion

The immune system consists of innate and adaptive immune systems that cooperatively protect the body from pathogenic microbes and toxins [10, 24]. The attack of pathogenic microbes and toxins activates innate immune cells such as macrophages, dendritic cells, and NK cells, which secrete interferon (IFN)-γ, TNF-α, IL-1β, IL-6, and IL-10 [4, 25]. These cytokines stimulate adaptive immune cells such as T cells, resulting in the differentiation of naïve T cells into Th1, Th2, Th17, and Treg cells [4, 25, 26]. The immunosuppression of host immune systems by stressors such as anticancer therapy cannot defend against pathogens and tumor progression. The hyperimmune responsiveness of host immune systems by pathogens, toxins, and allergens such as LPS excessively causes hypersensitivity including chronic inflammation through the activation of antigen-presenting cells and Th1/Treg cells [8, 26]. A wide imbalance in innate and adaptive immune systems perturbs gut microbiota, which can affect host systemic immune system through the regulation of the gut immune system [1, 3, 27]. Therefore, maintaining a delicate balance in the immune system by protecting against infection by pathogens and toxins is important for the body’s health.

In the present study, we found that oral administration of HDB1258, a novel probiotic isolated from healthy infant feces, significantly induced splenic NK cell cytotoxicity against YAC-1 tumor cells, peritoneal macrophage phagocytosis against *Candida albicans,* TNF-α to IL-10 expression ratio in the spleen, and TNF-α, IL-1β, and IL-6 expression in the colon,. Furthermore, HDB1258 increased the secretion of IgA into the feces, which serves as the first line of defense in protecting the gastrointestinal tract from pathogens and their toxins [28, 29]. However, it did not affect the differentiation of Th1 and Treg cells and expression of their transcription factors T-bet and Foxp3 in the systemic immune cell-representable spleen. These results suggest that HDB1258 may enhance the immune response by activating innate immunity including macrophage phagocytosis and NK cell cytotoxicity, not adaptive immunity, in a healthy host.

The intraperitoneal injection of LPS caused systemic inflammation: it induced the phagocytosis of peritoneal macrophages against *Candida albicans*, while the ratios of Treg to Th1 cell differentiation and Foxp3 to T-bet expression and IL-10 to TNF-α expression in the spleen and colon were suppressed. However, oral administration of HDB1258 increased the differentiation of Treg cells, not Th1 cells, while macrophage phagocytosis decreased. Furthermore, HDB1258 treatment significantly suppressed T-bet and TNF-α expression and induced the expression of IL-10 in the spleen, resulting in an increase in the ratios of Foxp3 to T-bet and IL-10 to TNF-α expression. Oral administration of HD1258 also suppressed LPS-induced colon shortening, myeloperoxidase activity, TNF-α, IL-1β, and IL-6 expression in the colon, while IL-10 expression increased. Furthermore, it increased the LPS-suppressed ratio of IL-10 to TNF-α expression. Villena et al. reported that *Lactobacillus rhamnosus* suppressed the immune response by regulating the expression of IL-10, an anti-inflammatory cytokine [30]. Nigar et al. reported that *Lactobacillus rhamnosus* potentiated the immune response by inducing the expression of IL-6 [31]. These results suggest that HDB1258 can suppress systemic inflammation including colitis by increasing the expression ratio of anti-inflammatory cytokines such as IL-10 to proinflammatory cytokines such as TNF-α.

In addition, Zhang et al. reported that *Lactobacillus rhamnosus* GG suppressed allergic airway inflammation in mice by inducing the Treg cell population, which was closely associated with gut microbiota composition [32]. Wang et al. reported that *Lactobacillus rhamnosus* GG enhanced TNF-α, IL-6, and IL-10 expression in gnotobiotic pigs vaccinated with an oral attenuated human rotavirus vaccine [33]. They suggested that *Lactobacillus rhamnosus* GG may regulate the homeostatic immune response by the modulation of gut microbiota. In the present study, we found that *Lactobacillus rhamnosus* enhanced the immune response in the healthy host by activating innate immune cells and suppressed the inflammatory response in the host with LPS-induced systemic inflammation by regulating innate and adaptive immune cells through the ratio of IL-10 to TNF-α expression. Moreover, oral administration of HDB1258 modified gut microbiota in mice with and without systemic inflammation. HDB1258 treatment also increased the Cyanobacteria and PAC000664_g (belonging to Bacteroidetes) populations and reduced Deferribacteres and EU622763_s group (belonging to Bacteroidetes) populations in mice with or without LPS-induced systemic inflammation. In particular, PAC001066_g and PAC001072_s populations showed a negative correlation with the ratio of IL-10 to TNF-α expression in the colon. PAC001070_s group population showed a positive correlation with the ratio of IL-10 to TNF-α expression in the colon of mice with or without LPS-induced systemic inflammation. PAC001127_g, and PAC001084_s, and PAC001756_s populations showed a negative correlation with the ratio of IL-10 to TNF-α expression in the spleen of mice with or without LPS-induced systemic inflammation. These cytokines are expressed in the gut immune cells such as macrophages, dendritic cells, and NK cells by the infection of pathogens into the gastrointestinal tract, resulting in the exclusion of pathogens in the host [4, 25]. However, if not excluded, chronic inflammation can be caused [25, 34]. Therefore, these immune-imbalanced diseases may be due to the hypoexpression of anti-inflammatory cytokines such as IL-10 or overexpression of proinflammatory cytokines such as TNF-α. Moreover, gut dysbiosis causes immune diseases including gastrointestinal inflammation [3, 35]. These results suggest that gut microbiota composition is closely associated with gut immune responses including the ratio of IL-10 to TNF-α expression and HDB1258 can regulate the immune responses by modulating the microbiota composition.

## Conclusions

HDB1258 may enhance the immune response by activating innate immunity including to macrophage phagocytosis and NK cell cytotoxicity, not the adaptive immunity, in the healthy host. HDB1258 can suppress systemic inflammation by increasing the expression ratio of anti-inflammatory cytokines such as IL-10 to proinflammatory cytokines such as TNF-α. HDB1258 may regulate the immune system including gut immune response by modulating the microbiota composition. Finally, HDB1258 may enforce the maintenance of a balanced immune response by the modulation of gut microbiota and IL-10 to TNF-α expression ratio in the immune cells.

## Materials and methods

### Materials

Sodium thioglycolate, 4′,6-diamidino-2-phenylindole, dilactate (DAPI), and RPMI 1640 were purchased from Sigma (St. Louis, MO). Enzyme-linked immunosorbent assay (ELISA) kits for IL-1β, IL-6, IL-10, and TNF-α were purchased from eBioscience (San Diego, CA). Antibodies were purchased from Cell Signaling Technology (Beverly, MA). CD4 T and NK cell isolation kits were purchased from Miltenyi Biotec (Teterow, Germany). A Vybrant CFDA SE Cell Tracer kit was purchased from Invitrogen (Grand Island, NY). A QIAamp DNA stool mini kit was purchased from Qiagen (Hiden, Germany).

### Culture of *Lactobacillus rhamnosus* HDB1258

HDB1258, isolated from breastfeeding infants (data not shown), was identified as *Lactobacillus rhamnosus* on the basis on the results of Gram staining, 16S rDNA sequencing (GenBank accession number MW193326), and API 50 CHL kit. *Lactobacillus rhamnosus* HDB1258 (HDB Cell Bank, Hyundae Bioland Co., Ltd., Korea) was inoculated into lava-seawater LAB media containing 8% glucose, 2% yeast extract, 0.5% soy peptone, 0.5% sodium acetate, 0.1% Tween 80, 0.01% MgSO_4_, 0.005% MnSO_4_, 0.2% potassium diphosphate, and 0.2% ammonium sulfate in 30% (v/v) lava-seawater (pH 6.5), incubated at 37°Cfor 20 h, and centrifuged (5000 g, 30 min) [36]. The resulting precipitate was mixed with hydroxypropyl methylcellulose and trehalose and freeze-dried. For the in vitro and in vivo experiments, it was suspended in saline and the number of viable HDB1258 was counted before used in experiment.

### Animals

C57BL/6 mice (male, 5 weeks old, 19 ~ 21 g) were supplied from Orient Bio (Seongnam-shi, Korea) and acclimatized for 7 days before the usage of experiments. All animals were maintained in the plastic cage with the 5 cm-raised wire floor under standard conditions (temperature, 20 ± 2 °C; humidity, 50 ± 10%, and lighting, 12 h/day). All mice were fed standard laboratory chow and tap water ad libitum. Animal experiments were conducted according to the NIH, University Guide for Laboratory Animal Care and Usage, and ARRIVE guidelines (https://arriveguidelines.org).

### Isolation and culture of macrophages

Macrophages, which were isolated from the peritoneal cavity of mice intraperitoneally injected with sodium thioglycolate according to the method of Jang et al. [17], were suspended in RPMI 1640 containing 10% fetal bovine serum and 1% antibiotics (RFA), seeded in 6-well plate, incubated at 37 °C for a day, and washed with RFA, as previously reported [14]. For the assay of IL-10 and TNF-α expression, macrophages were treated with LPS (80 ng/mL) in the presence or absence of HDB1258 (LL, 1 × 10^4^ CFU/mL; LH, 1 × 10^5^ CFU/mL)) for 20 h [17].

### Treatment with HDB1258, a probiotic, in mice with or without LPS-induced systemic inflammation

**Fig. 8:**
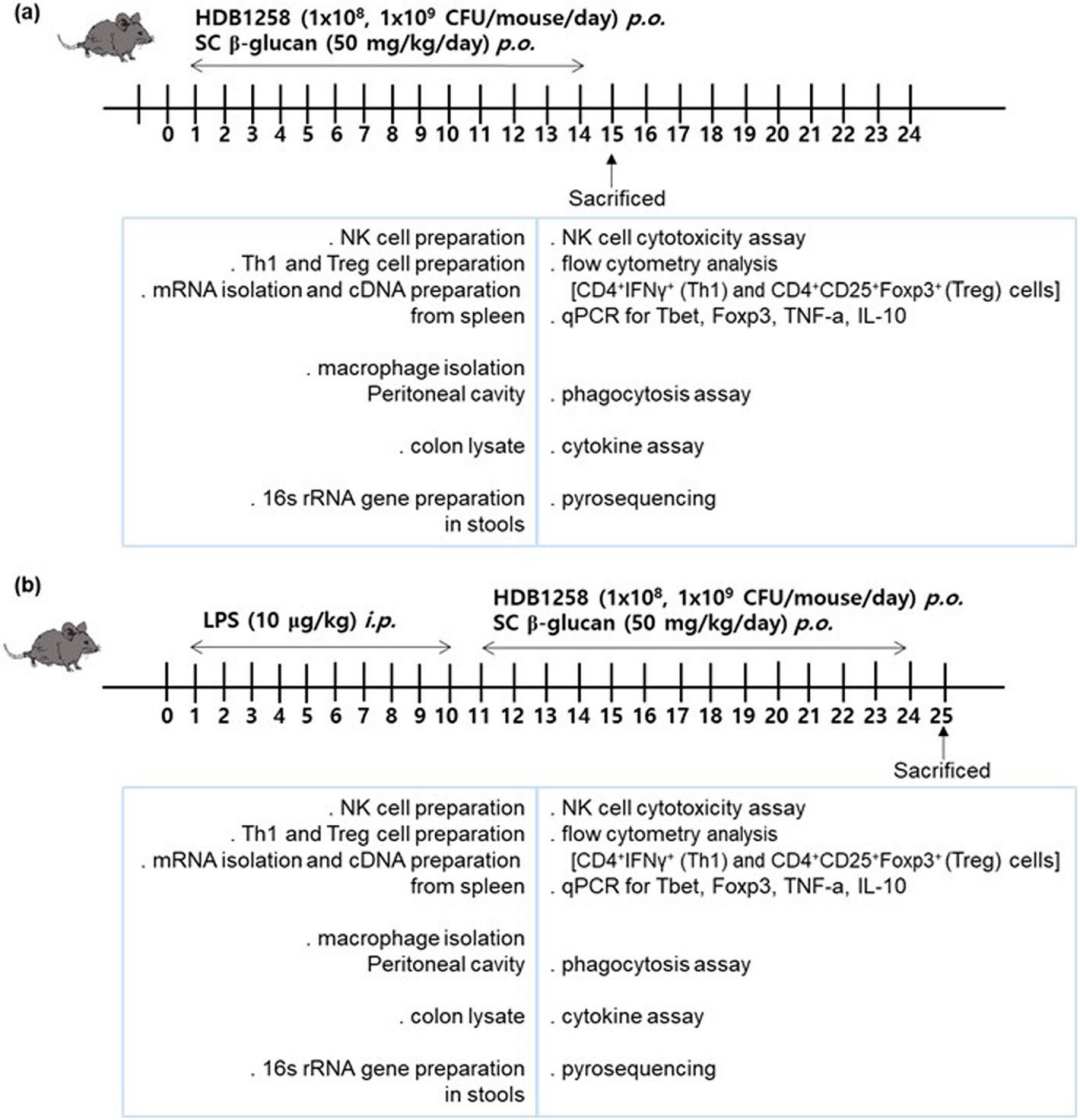
In vivo experimental schedules. **a** Experimental schedule in healthy control mice. **b** Experimental schedule in mice with LPS-induced systemic inflammation

To examine the immunomodulating effect of HDB1258, it was orally gavaged in mice with or without LPS-induced systemic inflammation. Normal control mice were orally gavaged with vehicle (saline) instead of HDB1258 (Fig. 8). Each group consisted of 7 mice. First, HDB1258 (LL, 1 × 10^8^ CFU/mouse/day; LH, 1 × 10^9^ CFU/mouse/day) or SC β-glucan (SCG, 50 mg/kg/day) was orally gavaged in control mice once a day for 14 days. Second, LPS (10 μg/kg, dissolved in 0.1 mL of saline) was intraperitoneally injected in mice once a day for 10 days according to the method of Jang et al. [15] and HDB1258 (LL, 1 × 10^8^ CFU/mouse/day; LH, 1 × 10^9^ CFU/mouse/day) was orally gavaged once a day for 14 days from next day after the final treatment with LPS. Mice were sacrificed 20 h after the final treatment with test agents by CO_2_ inhalation.

### Flow cytometric analysis of Th1 and Treg cells in the spleen

For the flow cytometric analysis of Th1 and Treg cells in the spleen, spleens were removed from mice, crushed, lysed with Tris-buffered ammonium chloride, suspended in RPMI 1640 medium, and then filtered. The CD4 T cells were isolated from the filtrates using a Pan T cell Isolation Kit II [37]. Isolated T cells were fixed and stained with anti-IFNγ or anti-Foxp3 antibodies. The stained cells (1 × 10^5^ cells) then analyzed by a flow cytometer (Accuri™ C6 Flow Cytometer, BD, Piscataway, NJ).

### Preparation of natural killer from splenocytes and its cytotoxicity assays

For the cytotoxic activity assay of splenic natural killer (NK) cells, NK cells were isolated from splenocytes prepared from the spleen of mice by using a NK cell isolation kit, as described previously [38]. The tumoricidal activity of the NK cells was evaluated by measuring the cytotoxicity against YAC-1 cells labeled with a Vybrant CFDA SE Cell Tracer kit according to the manufacturer’s protocol. NK cells (5 × 10^5^ per well) in the 96-well microplates were cultured with YAC-1 cells (5 × 10^5^ per well) for 24 h. The cells were washed and stained with propodium iodide and analyzed by a flow cytometer, as reported previously [38].

### Phagocytosis assay of peritoneal macrophages

Peritoneal macrophages were prepared as described previously [15]. Macrophage cells (1 × 10^6^ cell/well) was incubated with *Candida albicans* (1 × 10^4^ CFU/well, purchased from Korean Culture Center of Microorganisms (Seoul, Korea) in the 24-well microplates with complete RPMI 1640 medium and cultured for 24 h. The cultured supernatant (0.2 mL) was inoculated in Sabouraud dextrose agar for 24 h at 30 °C. The phagocytic activity (%) was indicated as [1-(the number of *Candida albicans* colonies grown in SDA per the number of *Candida albicans* initially incubated with macrophages)] × 100.

### Assay of myeloperoxidase activity

Myeloperoxidase activity was assayed according to the method of Jang et al. [15]. Colons were homogenized with cold RIPA lysis buffer and centrifuged at 10,000 *g* for 10 min. The supernatant was used as a crude enzyme solution. An aliquot (0.05 mL) of the supernatant was added in the reaction mixture (0.95 mL) containing 0.03% hydrogen peroxide and 1.6 mM tetramethylbenzidine. The absorbance at 650 nm time was monitored over 5 min. Activity was defined as the quantity degrading 1 μmol/mL of peroxide.

### Quantitative real time-polymerase chain reaction (qPCR)

For the assay of cytokine expression levels in the spleen, the qPCR was performed according to Kim et al. [20]. Briefly, the spleen was removed, homogenized. Total RNA was isolated with RNeasy Mini Kit (Qiagen, Hilden, Germany). The first-strand cDNA was synthesized by PrimeScript II 1st strand cDNA synthesis kit (Takara Bio Inc., Kanagawa, Japan) according to manufacturer’s protocol. The qPCR for TNF-α, IL-10, T-bet, Foxp3, and β-actin was performed utilizing Takara thermal cycler, which used SYBR premix agents (Takara Bio Inc.): activation of DNA polymerase at 95 °C for 5 min and 45 cycles of amplification at 95 °C for 10 s and at 60 °C for 30 s [38, 39]. The normalized expression of the assayed genes (TNF-α, IL-10, Foxp3, T-bet, and β-actin: their primers are described in Table S7), with respect to β-actin, was computed for all samples by using the Microsoft Excel data spreadsheet.

### Elisa

Colon tissues were lysed with ice-cold lysis RIPA buffer containing 50 mM Tris–HCl (pH 8.0), 150 mM sodium chloride, 1.0% Igepal CA-63, 0.5% sodium deoxycholate, 0.1% sodium dodecyl sulfate (SDS), 1% phosphatase inhibitor cocktail and 1% protease inhibitor cocktail and were centrifuged (10,000 g, 4 **°C**, and 10 min) [17]. For the assay of cytokines, colon homogenate supernatants were transferred in 96-well plate and assayed using ELISA Kits.

### Determination of fecal IgA

The fresh feces (0.1 g) was collected in the colon of mice after the sacrifice, suspended in phosphate-buffered saline, vortexed at 4 °C for 20 min, and centrifuged (10 min, 16,000 *g,* and 4 °C). The resulting supernatants were used for the assay of IgA. The IgA level was determined using mouse IgA ELISA kit (Invitrogen, Carlsbad, USA).

### Immunofluorescence assay

Immunofluorescence assay was performed according to the method of Kim et al. [18]. Briefly, the colon was post-fixed with 4% paraformaldehyde, cytoprotected in 30% sucrose solution, freezed, and cut using a cryostat (Leica, Nussloch, Germany). The section was washed with phosphate-buffered saline, incubated with antibodies for NF-κB (1:100, Cell Signaling Technology, Beverly, MA: cat # 8242S) and CD11c (1:200, Abcam, Cambridge, UK: cat #ab33483) antibody overnight, and treated with the secondary antibody for 2 h. A secondary antibody conjugated with the Alexa Fluor 488 (1:200, Invitrogen) or Alexa Fluor 594 (1:200, Invitrogen) was incubated to visualize. Nuclei were stained with DAPI. The stained sections were observed using a confocal microscope (Nanoscope Systems, Inc., Daejeon, Korea).

### 16S rRNA gene pyrosequencing

The bacterial genomic DNA was extracted for the fresh feces of mice using a QIAamp DNA stool mini kit according to Kim et al. [18]. The genomic DNA was amplified using barcoded primers targeted the bacterial 16S rRNA V4 region gene. Each amplicon was sequenced using Illumina iSeq 100 (San Diego, CA). Functional genes was predicted using the phylogenetic investigation of communities by reconstruction of unobserved states (PICRUSt) [18, 40]. Linear discriminant analysis (LDA) and cladograms were pictured using the LDA effect size (LefSe) on Galaxy platform (https://huttenhower.sph.harvard.edu/galaxy/) [41].

### Statistical analysis

All data are indicated as the means ± standard deviation (SD) and conducted GraphPad Prism 8 (GraphPad Software, Inc., San Diego, CA, USA). The significance was analyzed by unpaired Student’s *t*-test (for myeloperoxidase activity) and Kruskal-Wallis test with Dunn’s post-hoc test for non-parametric analysis (for other experimental data) (*p* < 0.05).

## Supplementary Information

**Additional file 1: Table S1.** Primer sequences used in the present study. **Table S1.** Effect of HDB1258 on the gut microbiota composition at the family level in mice. **Table S2.** Effect of HDB1258 on the gut microbiota composition at the genus level in mice. **Table S3.** Effect of HDB1258 on the gut microbiota composition at the species level in mice. **Table S4.** Effect of HDB1258 on the gut microbiota composition at the family level in mice with LPS-induced systemic inflammation. **Table S5.** Effect of HDB1258 on the gut microbiota composition at the genus level in mice with LPS-induced systemic inflammation. **Table S6.** Effect of HDB1258 on the gut microbiota composition at the species level in mice with LPS-induced systemic inflammation. **Table S7.** Primer sequences used in the present study. **Figure S1.** Effect of probiotics isolated from the feces of breastfeeding infants on the TNF-α expression in macrophages. **Figure S2.** The correlation between gut microbiota (at the species level) and ratio of TNF-α to IL-10 expression in the healthy mice. **Figure S3.** The correlation between gut microbiota (at the species level) and ratio of TNF-α to IL-10 expression in mice with LPS-induced systemic inflammation

## Data Availability

Pyrosequencing reads were deposited in the short read archive of NCBI under accession number PRJNA678595.

## Abbreviations

IFN: Interferon
IL: Interleukin
LPS: Lipopolysaccharide
NK: Natural killer
OUT: Operational taxonomic unit
PCoA: Principal coordinate analysis
SC: *Saccharomyces cerevisiae*
Th: Helper T
TNF: Tumor necrosis factor
Treg: Regulatory T
