# Supplementary material for: Lactobacillus rhamnosus HDB1258 modulates gut microbiota-mediated immune response in mice with or without lipopolysaccharide-induced systemic inflammation
Source: BMC Microbiol. 2021 May 13;21:146. doi: 10.1186/s12866-021-02192-4 (PMC8120827; doi:10.1186/s12866-021-02192-4)
Supplement: Supplementary file 1 — Additional file 1: Table S1. Primer sequences used in the present study. Table S1. Effect of HDB1258 on the gut microbiota composition at the family level in mice. Table S2. Effect of HDB1258 on the gut microbiota composition at the genus level in mice. Table S3. Effect of HDB1258 on the gut microbiota composition at the species level in mice. Table S4. Effect of HDB1258 on the gut microbiota composition at the family level in mice with LPS-induced systemic inflammation. Table S5. Effect of HDB1258 on the gut microbiota composition at the genus level in mice with LPS-induced systemic inflammation. Table S6. Effect of HDB1258 on the gut microbiota composition at the species level in mice with LPS-induced systemic inflammation. Table S7. Primer sequences used in the present study. Figure S1. Effect of probiotics isolated from the feces of breastfeeding infants on the TNF-α expression in macrophages. Figure S2. The correlation between gut microbiota (at the species level) and ratio of TNF-α to IL-10 expression in the healthy mice. Figure S3. The correlation between gut microbiota (at the species level) and ratio of TNF-α to IL-10 expression in mice with LPS-induced systemic inflammation [file 12866_2021_2192_MOESM1_ESM.docx]

**[Supplementary information]**

***Lactobacillus rhamnosus* HDB1258 modulates gut microbiota-mediated immune response in mice with or without LPS-induced systemic inflammation**

Sang-Kap Han^1,#^, Yeon-Jeong Shin^1,#^, Dong-Yeon Lee^1^, Kyung Min Kim^2^, Seo-Jin Yang^2^, Du Seong Kim^2^, Ji-Whi Choi^2^, Seunghun Lee^2^ and Dong-Hyun Kim^1,*^

*^1^Neurobiota Research Center, College of Pharmacy, Kyung Hee University, Kyungheedae-ro, Dongdaemun-gu, Seoul 02447, Korea; ^2^HYUNDAI Bioland Co., Ltd., Ansan 15407, Korea*

**^#^**Equally contributed

*****Correspondence: [dhkim@khu.ac.kr](mailto:dhkim@khu.ac.kr)

Neurobiota Research Center, College of Pharmacy, Kyung Hee University,

Table S1. Effect of HDB1258 on the gut microbiota composition at the family level in mice

| Taxon | Composition (%) | | | |
| --- | --- | --- | --- | --- |
|  | NC | SCG | LL | LH |
| Muribaculaceae | 39.62 ± 21.97^a^ | 34.35 ± 3.45 | 38.79 ± 6.03 | 35.66 ± 15.38 |
| Lachnospiraceae | 22.65 ± 13.47 | 14.42 ± 3.66 | 12.44 ± 4.82 | 25.53 ± 15.56 |
| Prevotellaceae | 11.45 ± 8.70 | 21.73 ± 7.63^#^ | 13.06 ± 5.67 | 13.98 ± 8.15 |
| Lactobacillaceae | 7.03 ± 9.75 | 5.60 ± 5.53 | 5.03 ± 3.03 | 2.03 ± 1.71 |
| Ruminococcaceae | 5.48 ± 3.53 | 8.68 ± 1.81 | 6.82 ± 2.47 | 5.93 ± 2.72 |
| Erysipelotrichaceae | 3.25 ± 4.19 | 3.88 ± 3.99 | 6.43 ± 8.22 | 0.64 ± 0.60 |
| Bacteroidaceae | 2.69 ± 1.43 | 3.32 ± 1.34 | 5.49 ± 2.42^#^ | 4.94 ± 4.53 |
| Helicobacteraceae | 2.00 ± 2.21 | 0.79 ± 0.71 | 0.55 ± 0.39 | 1.98 ± 1.71 |
| Rikenellaceae | 1.41 ± 0.94 | 1.42 ± 0.29 | 1.90 ± 2.32 | 1.55 ± 0.81 |
| Desulfovibrionaceae | 1.05 ± 0.97 | 0.51 ± 0.20 | 0.29 ± 0.13 | 1.06 ± 0.84 |
| FR888536_f | 0.58 ± 0.74 | 1.22 ± 1.37 | 1.32 ± 1.36 | 1.51 ± 1.25 |
| Sutterellaceae | 0.55 ± 0.33 | 1.10 ± 0.86 | 0.51 ± 0.36 | 0.86 ± 0.77 |
| Porphyromonadaceae | 0.41 ± 0.58 | 0.50 ± 0.22 | 0.54 ± 0.19 | 1.08 ± 0.49^#^ |
| Dehalobacterium_f | 0.25 ± 0.14 | 0.21 ± 0.09 | 0.15 ± 0.04 | 0.25 ± 0.17 |
| Bifidobacteriaceae | 0.24 ± 0.25 | 0.75 ± 0.83 | 0.60 ± 0.73 | 0.10 ± 0.09 |
| AC160630_f | 0.23 ± 0.23 | 0.15 ± 0.07 | 0.86 ± 0.89 | 1.25 ± 1.16^#^ |
| Deferribacteraceae | 0.15 ± 0.25 | 0.06 ± 0.15 | 0.00 ± 0.01 | 0.09 ± 0.10 |
| Coriobacteriaceae | 0.15 ± 0.13 | 0.08 ± 0.05 | 0.15 ± 0.10 | 0.10 ± 0.05 |
| Akkermansiaceae | 0.14 ± 0.16 | 0.18 ± 0.13 | 4.01 ± 7.47 | 0.13 ± 0.08 |
| Christensenellaceae | 0.13 ± 0.06 | 0.21 ± 0.07^#^ | 0.22 ± 0.11 | 0.36 ± 0.17^#^ |
| Clostridiaceae | 0.10 ± 0.07 | 0.09 ± 0.07 | 0.07 ± 0.06 | 0.07 ± 0.06 |

^a^mean± SD.

^#^p<0.05 vs. Con.

Table S2. Effect of HDB1258 on the gut microbiota composition at the genus level in mice

| Taxon Name | Composition (%) | | | |
| --- | --- | --- | --- | --- |
|  | NC | SCG | LL | LH |
| PAC001068_g | 14.53 ± 9.62^a^ | 15.41 ± 4.18 | 18.25 ± 4.85 | 10.62 ± 4.98 |
| Lactobacillus | 7.02 ± 9.74 | 5.58 ± 5.48 | 5.03 ± 3.03 | 2.03 ± 1.71 |
| PAC000186_g | 6.00 ± 3.88 | 5.17 ± 2.68 | 5.15 ± 2.65 | 4.02 ± 2.75 |
| Prevotellaceae_uc | 5.89 ± 5.10 | 12.63 ± 3.55# | 4.90 ± 3.27 | 1.62 ± 1.35 |
| PAC001485_g | 3.65 ± 4.54 | 0.03 ± 0.02 | 0.02 ± 0.02 | 0.02 ± 0.01 |
| Prevotella | 3.33 ± 3.69 | 6.99 ± 3.07 | 5.61 ± 2.86 | 3.41 ± 1.66 |
| PAC001112_g | 3.24 ± 2.47 | 1.79 ± 0.28 | 2.96 ± 1.34 | 2.60 ± 2.09 |
| PAC000664_g | 3.21 ± 2.07 | 2.54 ± 2.16 | 2.19 ± 1.28 | 7.60 ± 7.61 |
| Muribaculum | 3.19 ± 3.97 | 1.81 ± 0.80 | 1.95 ± 1.91 | 3.19 ± 1.93 |
| Bacteroides | 2.69 ± 1.43 | 3.32 ± 1.34 | 5.49 ± 2.42 | 4.93 ± 4.52 |
| Paraprevotella | 2.22 ± 3.13 | 2.11 ± 1.73 | 2.34 ± 3.14 | 8.53 ± 5.95 |
| KE159538_g | 2.08 ± 1.974 | 1.96 ± 1.22 | 1.98 ± 2.89 | 3.89 ± 5.80 |
| Helicobacter | 2.00 ± 2.21 | 0.79 ± 0.71 | 0.55 ± 0.39 | 1.98 ± 1.71 |
| PAC000198_g | 1.94 ± 0.75 | 2.09 ± 0.66 | 2.62 ± 1.31 | 3.51 ± 1.59 |
| PAC002367_g | 1.84 ± 3.51 | 0.03 ± 0.05 | 0.07 ± 0.06 | 0.90 ± 1.05 |
| PAC001472_g | 1.76 ± 1.15 | 1.73 ± 0.98 | 1.34 ± 0.56 | 0.86 ± 0.71 |
| LLKB_g | 1.70 ± 2.12 | 0.14 ± 0.07 | 0.38 ± 0.31 | 0.35 ± 0.40 |
| Pseudoflavonifractor | 1.58 ± 1.16 | 1.48 ± 0.56 | 0.93 ± 0.47 | 2.01 ± 0.89 |
| Faecalibaculum | 1.45 ± 1.74 | 3.11 ± 3.52 | 2.74 ± 3.82 | 0.13 ± 0.08 |
| Eubacterium_g6 | 1.30 ± 1.86 | 0.18 ± 0.11 | 0.35 ± 0.58 | 0.27 ± 0.18 |
| Alistipes | 1.24 ± 0.90 | 1.13 ± 0.22 | 1.58 ± 1.97 | 1.32 ± 0.77 |

^a^mean± SD.

^#^p<0.05 vs. Con.

Table S3. Effect of HDB1258 on the gut microbiota composition at the species level in mice

| Taxon Name | Composition (%) | | | |
| --- | --- | --- | --- | --- |
|  | NC | SCG | LL | LH |
| PAC001070_s group | 6.79 ± 8.19^a^ | 3.08 ± 1.57 | 4.67 ± 4.47 | 0.58 ± 0.31 |
| Lactobacillus murinus group | 5.91 ± 9.02 | 3.71 ± 4.24 | 3.73 ± 2.84 | 1.50 ± 1.60 |
| EU622763_s group | 2.69 ± 3.08 | 6.01 ± 2.85 | 4.81 ± 2.62 | 2.59 ± 1.47 |
| EU505186_s | 2.66 ± 3.30 | 0.03 ± 0.02 | 0.02 ± 0.02 | 0.02 ± 0.01 |
| PAC001065_s group | 2.26 ± 2.43 | 1.34 ± 0.45 | 2.22 ± 0.73 | 1.27 ± 0.82 |
| FJ880724_s | 2.00 ± 2.72 | 1.97 ± 1.59 | 2.18 ± 2.91 | 7.73 ± 5.27# |
| PAC001068_g_uc | 1.90 ± 1.56 | 1.85 ± 0.87 | 1.95 ± 0.57 | 1.26 ± 0.85 |
| PAC002367_s | 1.82 ± 3.47 | 0.03 ± 0.05 | 0.07 ± 0.06 | 0.89 ± 1.05 |
| PAC001267_s | 1.82 ± 2.03 | 0.32 ± 0.17 | 0.27 ± 0.10 | 1.02 ± 0.89 |
| PAC001077_s | 1.76 ± 2.98 | 0.17 ± 0.28 | 1.07 ± 1.33 | 1.01 ± 0.74 |
| PAC001072_s | 1.76 ± 0.88 | 2.99 ± 0.68# | 4.47 ± 1.38# | 5.93 ± 2.73# |
| EF097112_s | 1.54 ± 1.46 | 0.70 ± 0.30 | 1.46 ± 0.64 | 0.98 ± 0.67 |
| PAC001064_s | 1.44 ± 1.11 | 0.56 ± 0.20 | 0.67 ± 0.29 | 1.35 ± 1.03 |
| Faecalibaculum rodentium | 1.43 ± 1.71 | 3.06 ± 3.45 | 2.72 ± 3.79 | 0.13 ± 0.08 |
| Helicobacter rodentium group | 1.38 ± 2.11 | 0.31 ± 0.30 | 0.33 ± 0.38 | 1.22 ± 1.04 |
| AB606242_s | 1.23 ± 0.11 | 0.25 ± 0.48 | 1.13 ± 1.26 | 4.48 ± 5.25 |
| AB626939_s | 1.16 ± 1.93 | 0.07 ± 0.06 | 0.27 ± 0.25 | 0.20 ± 0.37 |
| Bacteroides acidifaciens group | 1.13 ± 0.59 | 1.33 ± 0.63 | 1.98 ± 1.58 | 0.84 ± 0.62 |
| PAC001472_g_uc | 1.02 ± 1.16 | 0.49 ± 0.44 | 0.32 ± 0.17 | 0.14 ± 0.18 |
| PAC000186_g_uc | 0.96 ± 0.68 | 1.09 ± 0.75 | 0.85 ± 0.64 | 0.60 ± 0.44 |
| JQ083736_s | 0.96 ± 0.68 | 1.09 ± 0.75 | 0.85 ± 0.64 | 0.60 ± 0.44 |

^a^mean± SD.

^#^p<0.05 vs. Con.

Table S4. Effect of HDB1258 on the gut microbiota composition at the family level in mice with LPS-induced systemic inflammation

| Taxon Name | Composition (%) | | | |
| --- | --- | --- | --- | --- |
|  | NC | LPS | LL | LH |
| Muribaculaceae | 32.26 ± 7.84^a^ | 30.50 ± 8.81 | 33.71 ± 9.51 | 35.68 ± 8.99 |
| Lachnospiraceae | 21.98± 7.10 | 9.54 ± 2.66^#^ | 22.25 ± 9.41* | 19.35 ± 11.22* |
| Prevotellaceae | 18.13 ± 6.12 | 21.48 ± 3.32 | 21.56 ± 8.92 | 21.97 ± 7.20 |
| Ruminococcaceae | 8.83 ± 2.09 | 11.65 ± 3.22 | 7.02 ± 2.64* | 6.52 ± 2.01* |
| Lactobacillaceae | 5.28 ± 4.67 | 6.46 ± 3.38 | 3.20 ± 1.75* | 2.99 ± 3.32 |
| Bacteroidaceae | 3.73 ± 1.38 | 4.45 ± 1.54 | 4.05 ± 1.89 | 3.99 ± 1.46 |
| Rikenellaceae | 2.40 ± 1.27 | 1.17 ± 0.50^#^ | 1.52 ± 0.59 | 2.21 ± 0.63* |
| Helicobacteraceae | 1.32 ± 0.79 | 0.21 ± 0.11^#^ | 0.70 ± 0.32* | 1.65 ± 2.44 |
| Erysipelotrichaceae | 1.11 ± 0.93 | 9.88 ± 4.46^#^ | 1.42 ± 2.17* | 0.58 ± 0.62* |
| FR888536_f | 0.83 ± 0.98 | 1.02 ± 0.97 | 0.40 ± 0.18 | 1.39 ± 2.33 |
| Porphyromonadaceae | 0.69 ± 0.30 | 0.65 ± 0.24 | 0.79 ± 0.25 | 0.64 ± 0.29 |
| Desulfovibrionaceae | 0.67 ± 0.32 | 0.33 ± 0.12^#^ | 0.84 ± 0.52* | 0.69 ± 0.51* |
| Akkermansiaceae | 0.52 ± 0.88 | 0.07 ± 0.04 | 0.09 ± 0.07 | 0.49 ± 0.41* |
| Dehalobacterium_f | 0.31 ± 0.13 | 0.14 ± 0.10^#^ | 0.31 ± 0.25 | 0.23 ± 0.16 |
| Sutterellaceae | 0.31 ± 0.16 | 0.95 ± 0.30^#^ | 0.37 ± 0.40* | 0.41 ± 0.16* |
| AC160630_f | 0.26 ± 0.15 | 0.29 ± 0.21 | 0.57 ± 0.32 | 0.11 ± 0.06 |
| Christensenellaceae | 0.18 ± 0.10 | 0.22 ± 0.06 | 0.15 ± 0.06* | 0.15 ± 0.08 |
| Bifidobacteriaceae | 0.14 ± 0.21 | 0.35 ± 0.26 | 0.11 ± 0.14* | 0.04 ± 0.08* |
| Odoribacteraceae | 0.14 ± 0.07 | 0.09 ± 0.10 | 0.08 ± 0.04 | 0.31 ± 0.26 |
| Coriobacteriaceae | 0.13 ± 0.08 | 0.08 ± 0.02 | 0.18 ± 0.07* | 0.06 ± 0.04 |
| Clostridiaceae | 0.11 ± 0.07 | 0.15 ± 0.07 | 0.13 ± 0.07 | 0.12 ± 0.09 |

^a^mean± SD.

^#^p<0.05 vs. Con. *p<0.05 vs.LPS.

Table S5. Effect of HDB1258 on the gut microbiota composition at the genus level in mice with LPS-induced systemic inflammation

| Taxon Name | Composition (%) | | | |
| --- | --- | --- | --- | --- |
|  | NC | SCG | LL | LH |
| PAC001068_g | 13.47 ± 4.87^a^ | 11.04 ± 3.43 | 13.14 ± 3.86 | 11.09 ± 3.97 |
| Prevotellaceae_uc | 9.13 ± 5.60 | 9.87 ± 2.71 | 12.28 ± 6.51 | 16.62 ± 4.27* |
| PAC000186_g | 6.01 ± 3.94 | 5.33 ± 1.31 | 5.12 ± 2.55 | 5.00 ± 1.54 |
| Prevotella | 5.30 ± 2.49 | 7.97 ± 2.20 | 6.96 ± 3.42 | 4.82 ± 3.87 |
| Lactobacillus | 5.28 ± 4.66 | 6.45 ± 3.38 | 3.20 ± 1.76* | 2.98 ± 3.32 |
| KE159538_g | 4.55 ± 3.44 | 0.43 ± 0.51# | 4.23 ± 5.43 | 2.21 ± 1.54* |
| Bacteroides | 3.37 ± 1.37 | 4.45 ± 1.54 | 4.05 ± 1.89 | 3.97 ± 1.45 |
| Paraprevotella | 3.33 ± 3.82 | 3.64 ± 3.64 | 2.33 ± 3.70 | 0.53 ± 0.99* |
| PAC000198_g | 2.90 ± 1.49 | 2.96 ± 1.08 | 3.02 ± 0.82 | 3.56 ± 1.25 |
| PAC000664_g | 2.78 ± 1.40 | 1.78 ± 0.99 | 5.32 ± 5.49 | 3.28 ± 1.61 |
| PAC002367_g | 2.30 ± 2.14 | 0.82 ± 1.62 | 1.29 ± 3.20 | 0.47 ± 0.43 |
| Muribaculum | 2.26 ± 1.41 | 1.86 ± 1.09 | 2.20 ± 1.73 | 5.38 ± 1.97* |
| PAC001112_g | 2.17 ± 1.38 | 2.58 ± 0.74 | 2.04 ± 0.36 | 2.85 ± 0.73 |
| Pseudoflavonifractor | 2.09 ± 0.69 | 0.98 ± 0.29# | 1.71 ± 0.66* | 1.50 ± 0.69 |
| Alistipes | 1.96 ± 1.08 | 1.03 ± 0.52 | 1.15 ± 0.54 | 1.90 ± 0.68* |
| PAC000661_g | 1.78 ± 0.88 | 2.51 ± 1.87 | 0.79 ± 0.64* | 1.36 ± 0.56 |
| Ruminococcus | 1.40 ± 1.05 | 4.90 ± 1.55# | 2.13 ± 2.17* | 1.44 ± 1.98* |
| Helicobacter | 1.32 ± 0.79 | 0.21 ± 0.11# | 0.70 ± 0.32* | 1.65 ± 2.44 |
| Eubacterium_g23 | 1.17 ± 1.39 | 1.56 ± 0.92 | 0.42 ± 0.60* | 0.24 ± 0.22* |
| Oscillibacter | 1.07 ± 0.59 | 0.32 ± 0.13# | 0.81 ± 0.46* | 1.04 ± 0.58* |
| LLKB_g | 0.89 ± 1.20 | 0.59 ± 0.35 | 0.91 ± 1.12 | 1.04 ± 0.97 |

^a^mean± SD.

^#^p<0.05 vs. Con. *p<0.05 vs.LPS.

Table S6. Effect of HDB1258 on the gut microbiota composition at the species level in mice with LPS-induced systemic inflammation

| Taxon Name | Composition (%) | | | |
| --- | --- | --- | --- | --- |
|  | NC | SCG | LL | LH |
| PAC001070_s group | 3.93 ± 1.48^a^ | 2.13 ± 2.01 | 1.83 ± 1.64 | 1.69 ± 1.53 |
| EU622763_s group | 3.73 ± 1.58 | 6.73 ± 1.74# | 5.54 ± 2.95 | 3.94 ± 3.31 |
| Lactobacillus murinus group | 3.52 ± 2.86 | 5.92 ± 3.37 | 2.82 ± 1.68* | 1.22 ± 0.92* |
| FJ880724_s | 3.03 ± 3.31 | 3.36 ± 3.34 | 2.14 ± 3.30 | 0.51 ± 0.94 |
| PAC001072_s | 2.94 ± 1.23 | 3.01 ± 0.45 | 4.37 ± 0.55* | 3.62 ± 1.27 |
| PAC001696_s | 2.54 ± 3.22 | 0.02 ± 0.03 | 0.35 ± 0.82 | 0.27 ± 0.27* |
| PAC002367_s | 2.29 ± 2.14 | 0.82 ± 1.61 | 1.29 ± 3.19 | 0.47 ± 0.43 |
| PAC001065_s group | 2.18 ± 0.61 | 0.86 ± 0.41# | 1.84 ± 1.56 | 1.55 ± 0.76 |
| PAC001068_g_uc | 1.62 ± 0.91 | 0.90 ± 0.40 | 1.33 ± 0.64 | 1.14 ± 0.60 |
| PAC000198_s | 1.50 ± 1.56 | 0.39 ± 0.38 | 0.03 ± 0.05* | 0.00 ± 0.00* |
| PAC002450_s | 1.43 ± 1.13 | 1.35 ± 1.54 | 1.25 ± 0.77 | 0.01 ± 0.01* |
| Bacteroides acidifaciens group | 1.34 ± 0.83 | 1.51 ± 0.20 | 1.12 ± 0.52 | 0.54 ± 0.27* |
| PAC002447_s | 1.15 ± 2.93 | 0.04 ± 0.04 | 0.00 ± 0.00* | 0.00 ± 0.00 |
| PAC001084_s | 1.10 ± 0.87 | 0.54 ± 0.30 | 1.48 ± 1.06* | 1.80 ± 0.75* |
| EF097112_s | 1.05 ± 0.58 | 1.20 ± 0.76 | 0.81 ± 0.39 | 1.01 ±.0.38 |
| PAC001064_s | 1.02 ± 0.73 | 0.92 ± 0.50 | 1.18 ± 0.36 | 1.19 ± 0.66 |
| AB599946_s | 1.00 ± 0.59 | 1.09 ± 0.63 | 0.55 ± 0.38 | 2.18 ± 0.92* |
| Lactobacillus reuteri group | 0.98 ± 1.11 | 0.17 ± 0.05 | 0.23 ± 0.18 | 0.88 ± 1.30 |
| DQ777952_s | 0.94 ± 1.50 | 0.04 ± 0.04 | 0.11 ± 0.12 | 0.03 ± 0.04 |
| PAC001982_s | 0.94 ± 1.17 | 1.49 ± 0.94 | 0.39 ± 0.60* | 0.11 ± 0.12* |
| PAC000186_g_uc | 0.93 ± 0.86 | 0.99 ± 0.38 | 0.75 ± 0.46 | 0.76 ± 0.41 |

^a^mean± SD.

^#^p<0.05 vs. Con. *p<0.05 vs.LPS.

Table S7. Primer sequences used in the present study

| Gene | Primer | Sequence |
| --- | --- | --- |
| TNF-α | Forward | 5’-AGCCCACGTAGCAAACCACCAA-3’ |
|  | Reverse | 5’-ACACCCATTCCCTTCACAGAGCAAT-3’ |
| IL-10 | Forward | 5’-CAGCCGGGAAGACAATAACTG-3’ |
|  | Reverse | 5’-CCGCAGCTCTAGGAGCATGT-3’ |
| T-bet | Forward | 5’-CAACAACCCCTTTGCCAAAG-3’ |
|  | Reverse | 5’-TCCCCCAAGCAGTTGACAGT-3’ |
| Foxp3 | Forward | 5’-AGAAGCTGGGAGCTATGCAG-3’ |
|  | Reverse | 5’-GCTACGATGCAGCAAGCGC-3’ |
| β-actin | Forward | 5’-TGTCCACCTTCCAGCAGATGT-3’ |
|  | Reverse | 5’- AGCTCAGTAACAGTCCGCCTAGA -3’ |

**
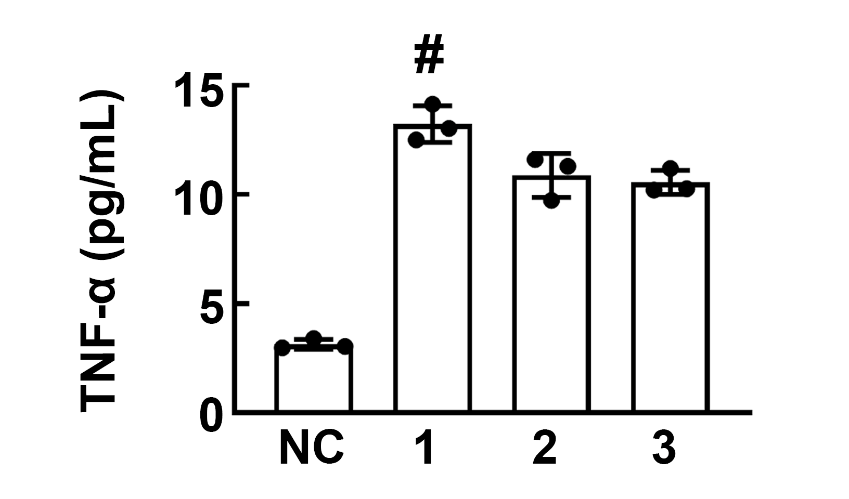
**

**Figure S1.** Effect of probiotics isolated from the feces of breastfeeding infants on the TNF-α expression in macrophages. Macrophage cells (1 × 10^6^/mL) isolated from peritoneal cavity were incubated with tested probiotics (1 × 10^5^ CFU/mL: 1, *Lactobacillus rhamnosus* HDB1258; 2, *Lactobacillus plantarum* HDB1234; 3, *Lactobacillus fermentum* HDB1096). Normal control group (NC) was treated with saline. Data values were described as mean ± SD (n = 3). ^*^*p* < 0.05 vs. NC.

**
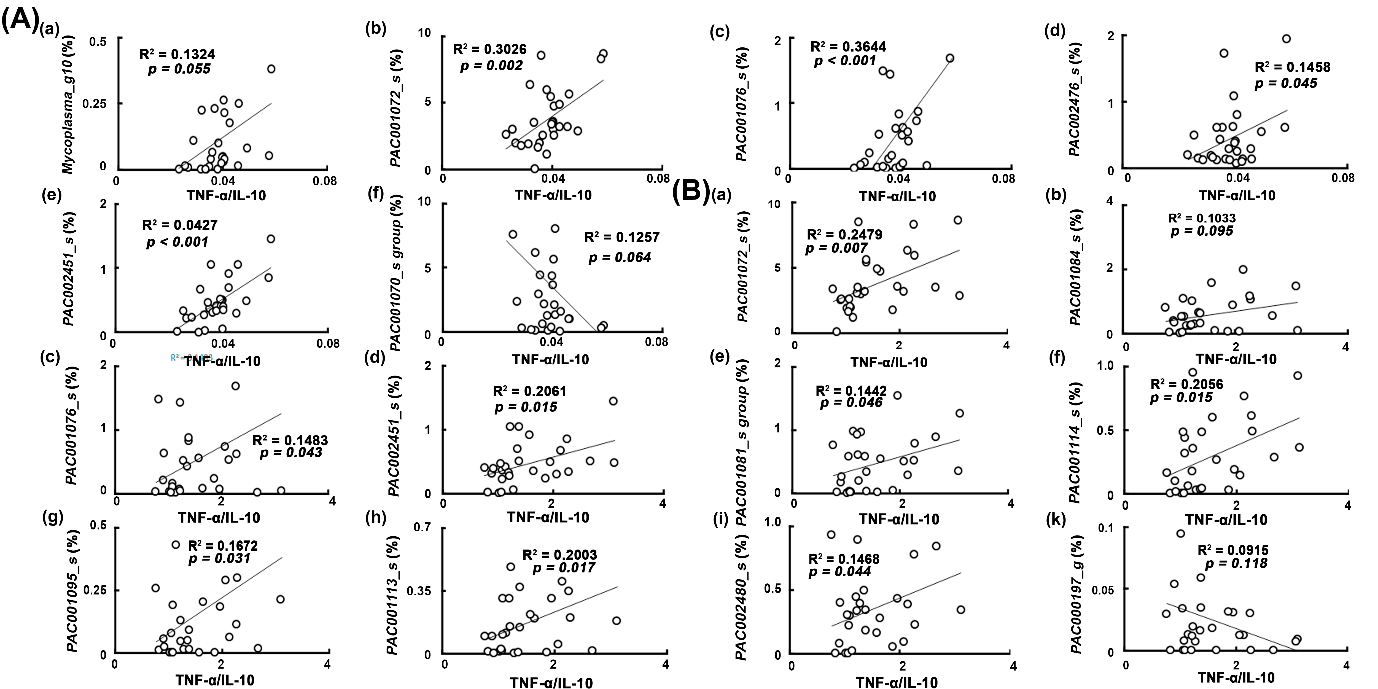
**

**Figure S2**. The correlation between gut microbiota (at the species level) and ratio of TNF-α to IL-10 expression in the healthy mice. (A) The correlation between gut microbiota (at the species level) and ratio of TNF-α to IL-10 expression in the colon. (E) The correlation between gut microbiota (at the species level) and TNF-α to IL-10 expression ratio in the spleen.

**
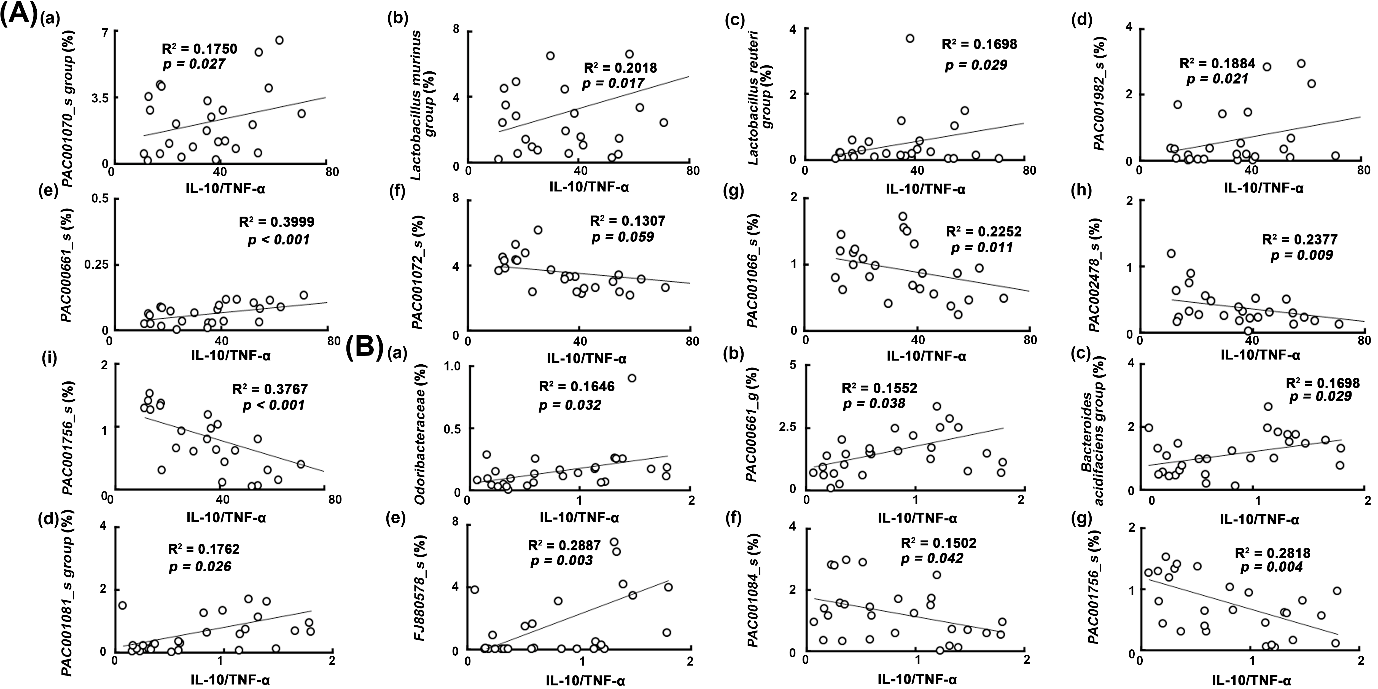
**

**Figure S3**. The correlation between gut microbiota (at the species level) and ratio of TNF-α to IL-10 expression in mice with LPS-induced systemic inflammation. (A) The correlation between gut microbiota (at the species level) and ratio of TNF-α to IL-10 expression in the colon. (E) The correlation between gut microbiota (at the species level) and TNF-α to IL-10 expression ratio in the spleen.
